# Enzymatic browning in fresh-cut fruits and vegetables: a comprehensive review of biochemical etiologies, detection techniques, and control strategies

**DOI:** 10.3389/fnut.2026.1948342

**Published:** 2026-10-14

**Authors:** Ali Hassan, Khubaib Ali, Waqar Mujahid, Hafiz Rizwan Sharif, Faizan Ul Haq, Muhammad Adnan Nasir, Imran Mahmood Khan, Waqas Ashraf

**Affiliations:** 1 National Institute of Food Science and Technology, University of Agriculture, Faisalabad, Pakistan; 2 College of Food Science and Engineering, Yangzhou University, Yangzhou, Jiangsu, China; 3 Institute of Food Science and Nutrition, University of Sargodha, Sargodha, Pakistan; 4 Department of Allied Health Sciences, The University of Chenab, Gujrat, Pakistan; 5 Nottingham Ningbo China Beacons of Excellence Research and Innovation Institute, University of Nottingham Ningbo China, Ningbo, China; 6 RII Centre for Molecular and Microbiome Sciences, Research Innovation Institute (RII), School of Health and Life Sciences, University of Nottingham Ningbo China, Ningbo, China; 7 School of Food Science and Engineering, Hainan University, Haikou, China

**Keywords:** browning indicators, control strategies, detection techniques, enzymatic browning, fresh-cut fruits and vegetables

## Abstract

Enzymatic browning causes substantial postharvest losses in fresh fruits and vegetables, yet the literature remains fragmented across polyphenol oxidase (PPO) biochemistry, detection technologies, and control strategies. This review synthesizes current knowledge across the browning continuum, emphasizing the emerging perspective that membrane degradation via the phospholipase D (PLD) and lipoxygenase (LOX) cascade may represent an important early event preceding PPO activation in specific physiological contexts, particularly senescence- and stress-associated browning. However, the relative contribution of membrane degradation versus direct enzyme-substrate contact varies among commodities and browning types, and membrane degradation is not always the sole or mandatory initiating event. A key mechanistic consideration is the distinction between soluble and membrane-bound PPO (sPPO and mPPO). While sPPO assays remain valuable for mechanistic screening, evidence from a limited number of comparative studies suggests they may not fully predict inhibitor performance in intact food matrices, and the extent of this limitation requires further investigation. The review also identifies a detection-intervention gap: while hyperspectral imaging, chlorophyll fluorescence, and electronic noses can detect early physiological changes associated with browning risk, validated protocols to respond to these signals remain underdeveloped. A conceptual framework linking detection to risk classification, decision thresholds, intervention, and verification is proposed to guide future research. Control strategies exhibit product-dependent efficacy, and a multi-hurdle integration model combining membrane preservation,

quinone trapping, and protective cultures is proposed as a hypothesis for future research rather than an established commercial strategy, requiring further validation to determine whether these components act synergistically.

## Graphical Abstract

**Figure:**
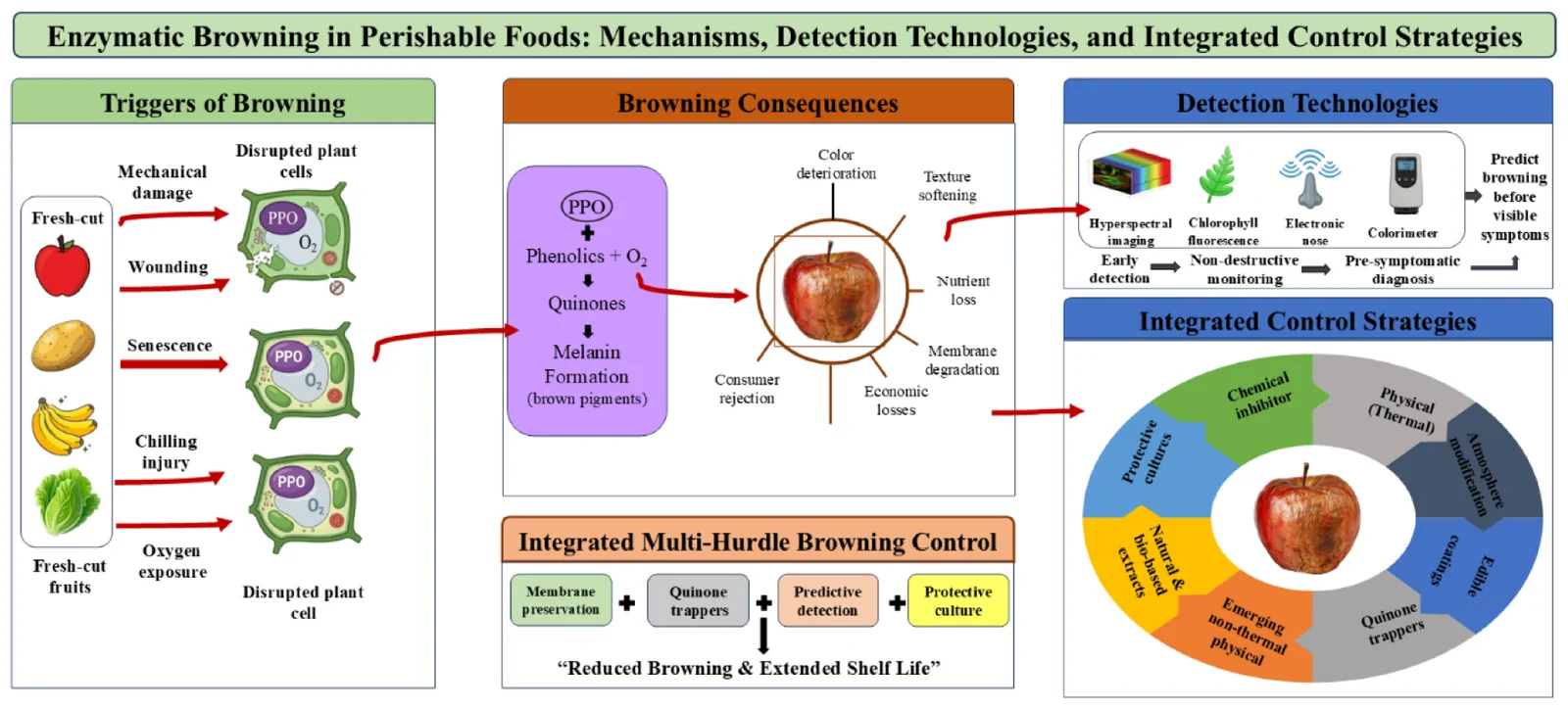
Causes, mechanisms, detection, and integrated control strategies of enzymatic browning in fresh-cut fruits and vegetables.

## 1 Introduction

Fruits and vegetables are essential components of the world’s food supply chain, providing vitamins and minerals to human diets and constituting a substantial economic sector, with annual international trade in fresh produce reaching $250 billion. But because of their natural perishability, they are particularly susceptible to postharvest losses, which, depending on the crop and location, account for 40%–55% of total production, particularly in developing countries (1). Enzymatic browning is one of the main mechanisms of quality loss among the different causes of deterioration. It is caused by mechanical damage during harvesting, handling, and processing, leading to cellular decompartmentalization and oxidation of phenolic compounds by polyphenol oxidase (PPO) enzymes (2). Molecular oxygen is activated by a binuclear copper core found in PPO. The hydroxylation of monophenols to o-diphenols and the oxidation of o-diphenols to very reactive o-quinones are the two consecutive processes that the activated oxygen complex catalyzes (3). Brown melanins are produced when these o-quinones polymerize non-enzymatically with proteins, amino acids, and other phenolics. Fresh-cut products exhibit an accelerated wound-induced browning response due to tissue disruption during processing, whereas whole commodities are affected by senescence-related membrane degradation during prolonged storage and by mechanical bruising from harvest and handling operations. This quality defect is present throughout the fresh produce supply chain (4). Consumer studies frequently show that customers reject damaged fruit even when it remains safe and nutritious because visual quality, especially color, is the key indicator of perceived freshness. Products that prioritize appearance as the primary purchasing factor are deemed unacceptable by consumers due to visual manifestations, ranging from superficial staining or peeling of sliced surfaces to internal flesh browning disease that develops without apparent symptoms (5, 6).

The phenolic substrates and enzymes of the PPO interact together to facilitate the oxidation processes involved in producing melanin. This response is not allowed in healthy tissues because of strict subcellular compartmentalization, such that PPO is found in plastids or chloroplasts, and phenolic chemicals are found in the vacuole (7). In many physiological contexts, browning is initiated when membrane integrity breaks down, allowing enzyme-substrate contact. However, the relative contribution of membrane degradation versus direct enzyme-substrate contact likely varies among commodities and browning types. For example, in fresh-cut tissue or severe mechanical damage, decompartmentalization occurs immediately, bringing PPO, phenolics, and oxygen into direct contact. Product-specific variations in phenolic substrates and PPO isoforms further influence substrate affinities and catalytic efficiencies based on plant species and tissue type (8). Thus, any factor that disrupts membrane integrity, such as mechanical damage, senescence, chilling injury, or pathogen attack, can initiate browning, regardless of the absolute concentration of PPO or phenolics. Fresh-cut products are more susceptible to browning through different physiological pathways. Fresh-cut products undergo a more intense wound response that includes immediate decompartmentalization, followed by transcriptional activation of phenylalanine ammonia-lyase (PAL). It promotes phenolic biosynthesis, and whole food products primarily undergo browning due to senescence-related membrane degradation during prolonged storage and mechanical bruising from harvest handling (9).

The long-standing traditional methods of controlling browning have been sulfites, organic acids, and cold storage (10). These approaches, however, are becoming less effective due to several challenges, including safety concerns about sulfite-induced allergic reactions, deterioration of sensory perception from acid-induced off-flavors, and customer aversion to synthetic additives in favor of clean-label products (11). These challenges resulted in a significant shift toward new technologies that meet the needs of consumers seeking natural and minimally processed foods. Currently, the popular ways of preservation are three complementary pathways: non-thermal physical methods such as cold plasma, pulsed electric fields, microwave, ultrasonication, and UV irradiation to maintain fresh-like qualities while inactivating enzymes; natural enzyme inhibitors based on plant extracts and agro-industrial products; and sustainable packaging approaches, including edible coating and active modified atmosphere systems to reduce plastic waste and prolong shelf life (12, 13).

Existing reviews addressing the topic of enzymatic browning have focused on just one aspect: PPO biochemistry; a single detection technology; enzymatic browning control strategy; or a specific commodity; and have not included the entire enzymatic browning continuum. Enzymatic browning research has long emphasized PPO as the primary driver, yet emerging evidence suggests that membrane degradation via phospholipase D (PLD) and lipoxygenase (LOX) may act as an important early event in certain browning contexts, particularly senescence- and stress-associated browning. However, the relative contribution of membrane degradation versus direct enzyme-substrate contact likely varies among commodities and physiological conditions. Screening with soluble PPO may overestimate inhibitor efficacy in certain food systems, while a detection-intervention gap persists; hyperspectral imaging (HSI) and related tools predict browning days in advance, but no validated protocols exist to act on these signals. These findings suggest that browning may be better understood as a context-dependent continuum in which membrane degradation and direct enzyme-substrate contact contribute to different extents depending on the commodity and physiological condition. This perspective suggests that a multi-hurdle approach combining membrane preservation, quinone trapping, and protective cultures may be a promising framework for future research. However, direct experimental evidence demonstrating synergy among these three components is currently lacking, and this strategy should be considered a hypothesis requiring validation rather than an established commercial approach. This review focuses primarily on enzymatic browning in fresh-cut fruits and vegetables, including sliced, diced, shredded, peeled, and trimmed commodities. Mushrooms are included as a minimally processed product highly susceptible to browning. Studies on whole commodities, juices, purees, and fermented systems are included selectively where they provide mechanistic insights or technology applications transferable to fresh-cut products; however, findings from liquid matrices should be extrapolated to solid fresh-cut tissues with caution. The term “perishable foods” is used broadly to refer to plant-based fresh commodities, but the specific focus of this review is fresh-cut fruits and vegetables. Meat, seafood, and dairy products are excluded because their browning mechanisms differ fundamentally from enzymatic browning in plant tissues; non-enzymatic browning predominates in animal-derived foods.

## 2 Literature search methodology

A structured literature search was conducted to identify relevant studies on the enzymatic browning of fresh-cut fruits and vegetables, covering biochemical mechanisms, detection technologies, and control strategies. The literature search was conducted between January 2000 and May 2026 to gather mechanistic, technological, and translational studies relevant to fresh-cut produce, with additional seminal references from prior years included to provide essential historical and foundational context, such as the biochemical characterization of polyphenol oxidase (PPO) and peroxidase (POD), the subcellular compartmentalization of PPO and phenolic substrates, and established mechanisms of enzymatic browning. The search was performed using the Scopus, Web of Science, PubMed, ScienceDirect, and Google Scholar databases. Search terms included combinations of keywords related to browning mechanisms, fresh-cut commodities, and detection and control technologies using Boolean operators (AND, OR). Core search terms included “enzymatic browning,” “polyphenol oxidase,” “PPO,” “peroxidase,” “POD,” “membrane degradation,” “phospholipase D,” “PLD,” “lipoxygenase,” “LOX,” and “phenolic metabolism,” combined with fresh-cut terms such as “fresh-cut,” “minimally processed,” “sliced,” “diced,” “shredded,” “peeled,” and “trimmed,” and with detection and control terms such as “hyperspectral imaging,” “chlorophyll fluorescence,” “electronic nose,” “cold plasma,” “pulsed electric field,” “ultrasound,” “high-pressure processing,” “ohmic heating,” “microwave,” “UV irradiation,” “edible coating,” “modified atmosphere packaging,” and “lactic acid bacteria.” A representative Boolean search string was: (“enzymatic browning” OR “polyphenol oxidase” OR “PPO” OR “peroxidase” OR “POD” OR “browning” OR “membrane degradation” OR “phospholipase D” OR “PLD” OR “lipoxygenase” OR “LOX” OR “phenolic metabolism”) AND (“fresh-cut” OR “minimally processed” OR “sliced” OR “diced” OR “shredded” OR “peeled” OR “trimmed”) AND (“detection” OR “hyperspectral” OR “multispectral” OR “chlorophyll fluorescence” OR “electronic nose” OR “control” OR “inhibition” OR “coating” OR “modified atmosphere” OR “non-thermal” OR “cold plasma” OR “pulsed electric field” OR “ultrasound” OR “high-pressure processing” OR “ohmic heating” OR “microwave” OR “UV” OR “edible coating” OR “protective culture” OR “lactic acid bacteria”). This string was adapted for each database with database-specific syntax adjustments. Duplicated and redundant search terms were removed and consolidated into standardized keyword groups.

Records obtained from different databases were consolidated, and duplicates were removed. The remaining articles were screened based on titles and abstracts, followed by full-text assessment for eligibility. Peer-reviewed research articles published in English and directly relevant to enzymatic browning in fresh-cut fruits and vegetables were included. Conference abstracts, patents, non-peer-reviewed publications, and studies focused on non-plant-based foods or processed foods with no relevance to fresh-cut commodities were excluded. Thermal and electrothermal treatments, such as blanching, ohmic heating, and microwave heating, were included where they were evaluated as anti-browning interventions for fresh-cut products or where they provided mechanistic insights into PPO/POD inactivation transferable to fresh-cut systems. Studies were excluded if they focused exclusively on thermal processing for purposes unrelated to enzymatic browning, such as sterilization or pasteurization of liquid foods without browning assessment, or if they addressed non-enzymatic browning without relevance to fresh-cut produce. The initial search yielded 712 records. After removal of duplicates (*n* = 204), 508 records were screened by title and abstract. Of these, 198 records were excluded because they did not address enzymatic browning, were not peer-reviewed, or focused on non-plant-based foods. The remaining 310 full-text articles were assessed for eligibility, of which 154 were excluded for insufficient methodological detail (*n* = 52), focus on thermal processing unrelated to browning or on non-enzymatic browning (*n* = 36), non-plant-based or processed foods with no relevance to fresh-cut commodities (*n* = 28), lack of quantitative data (*n* = 22), or non-English language (*n* = 16). A total of 156 studies met the inclusion criteria and were included in the final synthesis.

Given the broad and multidisciplinary nature of the topic, this review adopts a structured narrative approach that combines systematic literature searching with critical synthesis of evidence. The included studies vary in their experimental approaches, ranging from purified enzyme assays to food matrix evaluations and storage trials. To facilitate interpretation, studies are described with explicit reference to the system used, such as purified PPO, soluble PPO extract, membrane-bound PPO, model system, fresh-cut food matrix, or storage trial. Where findings are extrapolated beyond the system in which they were obtained, this is stated explicitly. The majority of studies reviewed here used purified enzyme assays or soluble PPO extracts, while relatively fewer studies employed membrane-bound PPO or food matrices, and pilot-scale or industrial validation studies are scarce. A formal meta-analysis was not conducted because of the heterogeneity of study designs and outcome measures; instead, a qualitative synthesis was performed, and findings from studies with stronger methodological rigor were prioritized.

## 3 Causes of enzymatic browning in fresh-cut fruits and vegetables

### 3.1 Enzymes (PPO, POD) and substrates

Enzymatic browning is primarily driven by the interaction of three essential components: oxidative enzymes, phenolic substrates, and molecular oxygen. PPOs are copper-containing enzymes that catalyze two sequential reactions: hydroxylation of monophenols to o-diphenols and oxidation of o-diphenols to reactive o-quinones (14). Brown melanins are formed when these o-quinones, which are electrophilic intermediates, non-enzymatically polymerize with proteins, amino acids, and other phenolics. Phenolic substrates reside in vacuoles in intact tissues, where PPO is segregated within plastids to prevent enzyme-substrate contact (2). In the presence of hydrogen peroxide (H_2_O_2_), POD can also oxidize phenolic compounds to quinones, thereby exacerbating PPO-induced browning. However, each enzyme’s contribution differs greatly among commodities. The main cause of browning in apples is PPO activity; the browning-sensitive variety “Fuji” has far higher PPO activity and a stronger substrate affinity than the resistant line (15). After 8 days of storage, the combination treatment decreased PPO and POD activity in fresh-cut potatoes by 35.1% and 55.7%, respectively, suggesting that both enzymes significantly contribute to browning in this product (16). In contrast, white button mushrooms treated with pulsed light showed PPO and POD inactivation rate constants of 3.84 and 2.55 cm^2^/J, respectively, indicating similar enzyme contributions (17). Generalized browning models frequently overlook this interspecific variability in substrate preference and enzyme expression.

Phenylalanine ammonia-lyase is the entry-point and rate-limiting enzyme in the phenylpropanoid pathway, catalyzing the deamination of L-phenylalanine to trans-cinnamic acid (18). Cinnamate 4-hydroxylase (C4H) and 4-coumaroyl-CoA ligase (4CL) are involved in the pathway’s sequential processes, which result in the formation of several phenolic compounds, such as flavonoids and chlorogenic acid (18, 19). Fresh-cut processing wounds rapidly upregulate PAL transcription, resulting in the de novo production of phenolic compounds that serve as substrates for enzymatic oxidation. Unlike quick browning caused by pre-existing phenolics, this wound-induced phenolic accumulation causes delayed-type browning, which appears hours to days after cutting. For browning regulation, the timing of PAL induction is crucial (20, 21). Phenolic substrates are physically isolated from PPO, which is found in plastids, and are contained within the vacuole in intact tissues. Enzyme-substrate interaction and subsequent oxidation are made possible by the disruption of this compartmentalization caused by wounding or membrane disintegration (22).

In enzymatic browning, POD demonstrates two functions. By employing H2O_2_ as a co-substrate to oxidize phenolic compounds to quinones, POD aids in browning (23). On the other hand, POD also helps metabolize ROS, especially Class I heme POD, which scavenges H2O_2_ to preserve membrane integrity (24). The balance between these pro-oxidant and antioxidant activities, which is influenced by particular isoenzymes, subcellular location, and substrate specificity, determines POD’s net contribution (25). In fresh-cut products, wound-induced H_2_O_2_ production often shifts the balance toward pro-oxidant activity, making POD a contributor to browning (26). This dual functionality explains why POD inactivation does not always correlate with reduced browning, as inhibiting POD may simultaneously impair H_2_O_2_ detoxification (23).

### 3.2 Expression of PPO and PAL genes in response to stress

A key regulatory mechanism in the browning response of fresh-cut products following mechanical damage is the transcriptional regulation of PPO and PAL genes. A comprehensive study of PPO gene families across horticultural crops has been made possible by advances in genomics, revealing novel complexity in which single genes have given way to multigene families with varied expression patterns and potential functional specialization (27). Guo et al. (28) identified 17 members of the LsPPO gene family in lettuce and their expression patterns under wound stress. It was concluded that fresh-cut processing led to a significant upregulation of most LsPPO members. Transcriptional activation and functional enzyme activity are defined by a crucial quantitative duration. Transcriptomic analysis of apple pulp identified 1,448 differentially expressed genes within 30–60 min of cutting, with 77 DEGs shared across all browning phenotypes at 60 min. These genes were associated with a conserved regulatory network comprising calcium-binding proteins, heat-shock proteins, redox-responsive transcription factors, and WRKY family transcription factors (29). However, the causal relationships between these transcriptional changes and browning development remain to be established.

Recently, research has moved beyond transcriptional analysis alone by integrating multi-omics methods. Yuan et al. (5) examined existing multi-omics approaches, such as transcriptomic, proteomic, and metabolomic techniques, to clarify browning mechanisms and find possible metabolic indicators and mitigation pathways. GABA treatment prevented browning in fresh-cut potatoes and was associated with changes in the expression of 3,625 genes, with a notable enrichment in phenylpropanoid biosynthesis, along with concurrent modifications in PPO activity and phenolic metabolism (30). However, these findings demonstrate association rather than causation, and the specific contribution of individual gene expression changes to browning reduction requires further investigation. The delay between transcriptional activation and the production of functional enzymes may have important implications for control: it can be hypothesized that PAL inhibitors would need to be present during this early phase to be effective, whereas most postharvest treatments are applied immediately after cutting. However, direct experimental evidence for this timing requirement is currently lacking, and this remains a proposed implication rather than an established conclusion. The biphasic character of the wound response, immediate oxidation of pre-existing phenolics followed by delayed synthesis of new substrates, indicates that multi-phase interventions, as compared to single-point treatments, may be essential to effective control (31). CRISPR/Cas9 editing of LcPPO in litchi callus confirmed that PPO is functionally required for browning, as edited lines showed lower PPO expression, reduced activity, and slower browning (32). In conclusion, Acute browning is poorly addressed by gene expression studies because of transcriptional delays; integrated omics and gene editing show promise, but minute-scale interventions are still required.

### 3.3 Physiological factors

Wounding, mechanical damage, and senescence are the three physiological mechanisms that contribute to enzymatic browning. Wounding, which usually occurs during fresh-cut processing, is defined as tissue injury accompanied by a break in the skin or surface that results in quick decompartmentalization and wound signaling (33, 34). In contrast, mechanical damage describes changes in an intact product’s appearance or shape brought on by physical forces without necessarily breaking the surface. This frequently results in internal bruising that takes hours to days to develop (4, 35).

#### 3.3.1 Wounding

Wounding caused by fresh-cut processing triggers a complex physiological chain reaction that extends beyond simple cellular death. Cutting or peeling plant tissues fundamentally undermines their structural integrity by disrupting subcellular compartmentalization, which typically restricts PPO and POD inside plastids and peroxisomes and sequesters phenolic substrates within vacuoles (5). This decompartmentalization allows enzyme-substrate interaction in the presence of ambient oxygen, leading to rapid enzymatic oxidation and immediate browning at the sliced surface. However, as Murata (31) explained, this immediate browning is only the first stage of the wound response. An important distinction exists between “delayed-type” browning, which occurs in foods like lettuce and mung bean sprouts, where visible discoloration appears only hours or days after the synthesis of phenolic compounds. Therefore, regulating PAL induction is required to prevent delayed-type browning, whereas regulating PPO’s oxidative polymerization of phenolic compounds is essential for quick browning. This mechanism was further examined by Chen et al. (35) in fresh-cut “Fuji” apples, contrasting the reactions of inner and outer tissues to mechanical trauma. Surface tissue had significantly higher levels of lipid peroxidation, as evidenced by increased malondialdehyde concentration and increased activities of PPO, POD, PAL, and LOX. These findings reveal considerable spatial and temporal heterogeneity in wound-induced browning responses, complicating both prediction and intervention.

Wounding from fresh-cut processing disrupts phytohormone homeostasis, triggering signaling cascades that amplify browning. Ethylene is rapidly synthesized in wounded tissues and accelerates senescence, promotes membrane lipid degradation, and upregulates PAL and PPO gene expression. However, wound-induced ethylene has a negligible effect on phenolic metabolism in lettuce, indicating that wounding and ethylene act independently in some commodities (36, 37). In stress signaling, methyl jasmonate (MeJA) functions as a second messenger. In fresh-cut potato cubes, MeJA treatment enhanced PPO, POD, and CAT gene expression as well as PAL, C4H, and 4CL activity. MeJA improved wound-induced phenolic buildup in pitaya fruit by controlling energy status and sugar content. In certain instances, MeJA treatment increased browning even while it promoted healing of wounds. The reaction to MeJA is very species- and tissue-dependent (26, 38).

#### 3.3.2 Senescence and membrane degradation

In the fresh-cut products discussed in the cited studies, slow browning is primarily attributed to senescence-driven membrane breakdown, whereas fast browning is associated with wounding. Whether this pattern applies universally across all whole perishable products requires further investigation. Membrane lipid degradation involves a coordinated enzymatic cascade rather than a single step. PLD hydrolyzes phospholipids to phosphatidic acid (PA) and a free head group. Phosphatidic acid phosphatase (PAP) then dephosphorylates PA to diacylglycerol (DAG), which is subsequently hydrolyzed by DAG lipases to release free polyunsaturated fatty acids (PUFAs). Alternatively, phospholipase A_2_ (PLA_2_) can directly release free fatty acids from phospholipids. The liberated PUFAs, particularly linoleic and β-linolenic acids, serve as substrates for LOX, which catalyzes their oxygenation to hydroperoxides. These hydroperoxides are further metabolized to oxylipins and volatile aldehydes, contributing to membrane destabilization and cellular decompartmentalization (38). Wang et al. (39) proposed membrane catabolism as an equally early interactive system, and the argument against phenolic oxidation was demonstrated by demonstrating that 10 out of 11 of the accumulated metabolites leading to browning were lipid-derived. Bridging wounding and senescence mechanisms, Ru et al. (40) demonstrated that fresh-cut stem lettuce is prevented from browning by β-membrane-protecting treatments through the inhibition of LOX/PLD activities and suppression of membrane lipid metabolism genes. Zhong et al. (41) hypothesized that signaling molecules like nitric oxide specifically inhibit postharvest senescence by acting via pathways that can be summarized as reducing membrane lipid peroxidation and preserving membrane integrity, with membrane preservation being critical in quality management. As membrane-targeting studies have never been compared with classic PPO inhibitors under controlled conditions, it remains unclear whether preserving membrane integrity alone is sufficient to prevent browning at elevated levels of PPO/phenolic compounds. The importance of membrane integrity as a determinant of browning susceptibility was demonstrated by Li et al. (42), who showed that membrane degradation precedes phenolic oxidation in fresh-cut pear. This finding provided foundational evidence that membrane preservation may be as important as enzyme inhibition for browning control.

In reaction to injury and stress, reactive oxygen species (ROS) such as superoxide anion (O_2_^–^), H_2_O_2_, and hydroxyl radicals (•OH) are formed quickly. Enzymatic antioxidants and non-enzymatic antioxidants (ascorbic acid, glutathione, phenolics) keep ROS levels low under physiological conditions (23). When ROS production exceeds scavenging capacity, oxidative stress leads to membrane lipid peroxidation and cellular decompartmentalization (43). ROS and browning interact reciprocally. ROS-induced membrane breakdown facilitates the PPO-phenolic interaction. On the other hand, during phenolic oxidation, PPO produces ROS, resulting in a positive feedback loop. By suppressing the expression of ACC oxidase and ACC synthase, melatonin treatment lowers ethylene production, delays senescence, and reduces membrane lipid peroxidation, thereby preserving membrane integrity (44, 45). Fresh-cut produce’s browning processes vary depending on the situation. Senescence-related browning, which facilitates PPO-phenolic interaction, develops gradually over extended storage and results in greater membrane deterioration. Wounding from processing causes immediate decompartmentalization at cut surfaces, followed by delayed PAL-mediated phenolic synthesis (34, 46). Chilling injury in cold-stored commodities manifests as internal flesh browning, pitting, and water-soaked lesions, driven by membrane lipid peroxidation and oxidative stress (46). Impact or compression bruises damage subcutaneous tissue without breaking the skin, and browning appears hours to days later (4). Internal physiological disorders, such as CO_2_ injury in pears and internal browning in pineapples, involve metabolic dysregulation and membrane disruption that may occur without external symptoms (47, 48). The need for context-specific strategies for intervention is highlighted by these different paths.

#### 3.3.3 Mechanical damage

Mechanical damage causes thirty to forty percent of all post-harvest losses between harvest and consumption. These are also external stimuli that trigger wounding and senescence pathways (4). Xu et al. (34) investigated the formation of bruises in Red Delicious apples. They found that a specific area of cell death in the injured pulp expands laterally. Damage peaks at 30 min following impact and levels off by 72 min; about 60 percent of tissue injury occurs in the core, and 40 percent toward the pericarp. Although this temporal characterization is complete, no model connects impact force to the ultimate degree of browning. A similar pattern emerged in sweet potatoes. Zhang et al. (49) found that an extrusion pressure of 180 N resulted in higher porosity, cellular breakage, starch aggregation, weight loss, and a 21.08% reduction in hardness after 6 days of storage. Once again, damage is quantified, though the mechanical factors that produce browning cannot be predicted or determined. Yuan et al. (5) established a link between mechanical stress and membrane degradation by demonstrating that mechanical damage activates phospholipase (PL) and LOX, which catalyze membrane lipid degradation and peroxidation, respectively. Primary physical damage and subsequent biochemical deterioration are the two stages of mechanical damage. The main occurrence is the initial compromise of membrane integrity due to direct mechanical rupture of cellular membranes brought on by impact, compression, or vibration. A subsequent metabolic reaction involving the activation of LOX and PLD is brought on by this physical damage. Zhao et al. (50) demonstrated that after mechanical injury, PLD and LOX activities increase together with increased gene expression, indicating that the enzymatic cascade is a result of the physical damage signal rather than its source. Therefore, PLD/LOX-mediated membrane degradation is a component of the subsequent biochemical response that follows the initial physical rupture of membrane integrity, even though it aids in the development of browning.

Chilling injury, a distinct physiological disorder caused by low-temperature stress, also involves membrane lipid degradation. Wang et al. (51) demonstrated that an imbalance between flavonoid depletion and lipid metabolism may be a key mechanism of chilling injury in cold-stored cucumbers, highlighting the interplay between phenolic metabolism and membrane degradation under cold stress. Rashvand et al. (52) demonstrated a clear correlation between mechanical damage, fruit maturity at harvest, and loss of nutritional quality by showing that bruise volume increased with fruit maturity across three olive varietals. Nonetheless, maturity is rarely a predictive characteristic in forecasting models. The lack of predictive modeling and regular damage evaluation is a significant disadvantage. Cross-study comparisons are not possible because different studies employ various impact methodologies. The severity of browning cannot currently be determined mathematically from mechanical parameters and product attributes. The browning triad, PPO, phenolics, and oxygen, is presented in Figure 1. Until in cell damage allows mixing, browning is prevented by their compartmentalization. Crucially, this trivalent framework lacks isoform-specific reactions and the membrane-bound nature of active PPO, both of which are major causes of inhibitor degradation in real food matrices.

**FIGURE 1:**
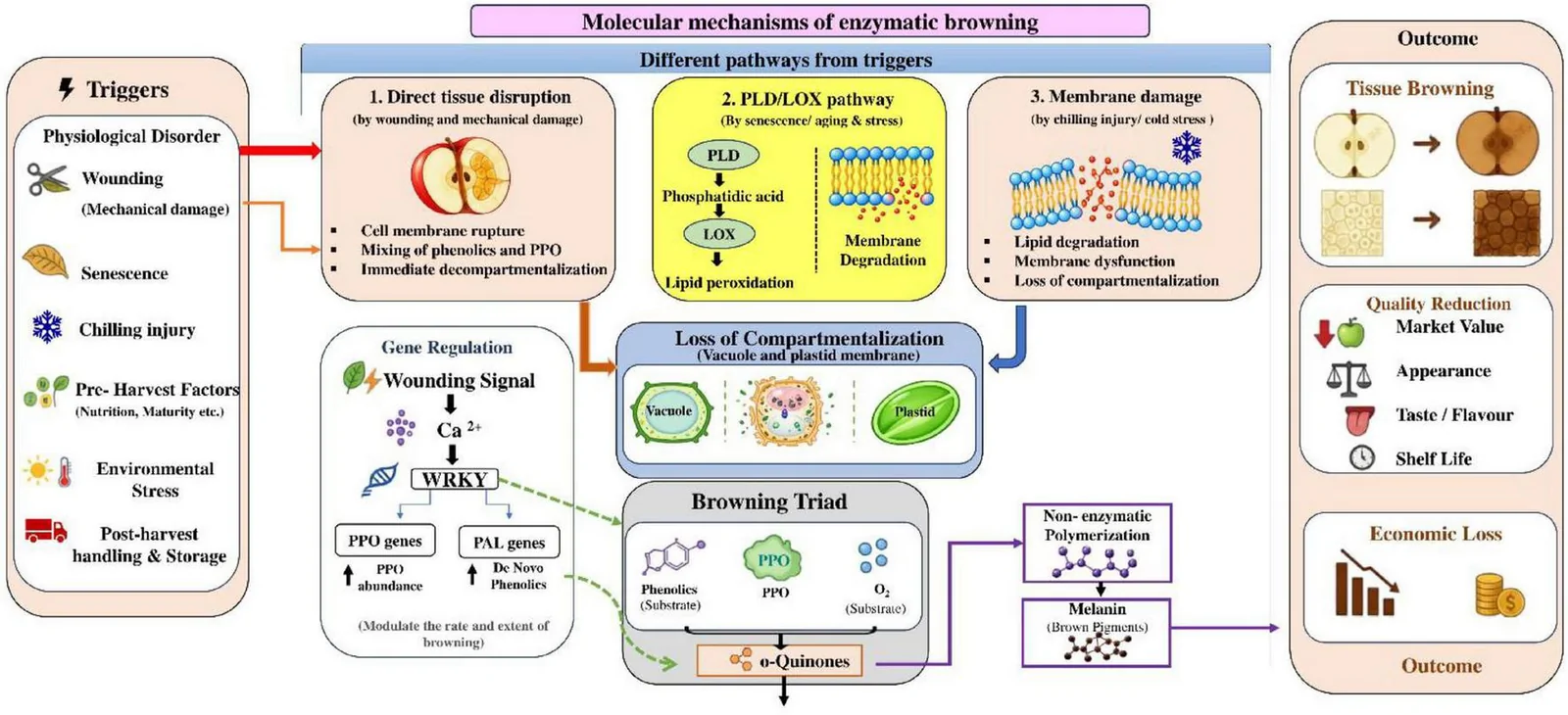
Mechanistic pathway of enzymatic browning.

## 4 Detection technologies for browning

The non-destructive methods described here differ in specificity and can be categorized as direct browning indicators or biochemical precursors. Colorimetric techniques directly quantify surface discoloration. In contrast, spectral imaging, chlorophyll fluorescence (CF), and electronic noses (E-nose) measure changes associated with browning risk without confirming PPO-mediated browning. For example, Fv/Fm decline indicates photosynthetic stress, not browning, and Volatile Organic Compound (VOC) profiles may reflect microbial activity or aging. Precursor signals should therefore be interpreted as indicators of browning risk rather than direct measures of browning. Each technology is described below with its specific metric, sensitivity, limitations, and technology readiness level.

### 4.1 Hyperspectral and multispectral imaging

Non-destructive optical methods, such as HSI and MSI, use spectral changes that precede visible deterioration to detect enzymatic browning. Three phenomena contribute to the mechanistic basis. First, as the chlorogenic acid oxidizes to quinones, the absorbance in the UV-Vis spectrum at 300–500 nm changes. Second, light scattering at cellular interfaces increases with membrane breakdown. Third, water redistribution in tissue injury produces SWIR signals at 1,400–1,500 nm that correlate with O-H stretching. This explains why browning is anticipated in HSI days before symptoms manifest. The technique does not recognize the discoloration but investigates prior physicochemical events (53). Zhang et al. (54) detected apple bruises two to six hours post-wounding with deep learning and SWIR data. By applying a pseudo-color transformation to enhance the contrast between damaged and healthy tissues, the YOLOv5 model achieved 130 frames per second and 95% recognition accuracy. Industrial application, however, is limited by three challenges. These are costly cameras; the computing needs of a workstation for GPU-based workloads to process real-time data; and the appearance of bruises, which vary by cultivar, meaning models must be trained per cultivar. In addition to detecting bruises, HSI has been expanded to forecast biochemical indicators of browning susceptibility. Park et al. (55) characterized the blanched potato slices using imaging with the Hyperspectral microscope (HMI) and HSI to develop predictive models of PPO activity and total phenolic content, with R_2_ values between 0.71 and 0.87. The multiscale application of HSI for browning detection is illustrated in Figure 2. RGB images (Figure 2A) show that insufficient blanching causes strong localized browning, while HSI (Figure 2B) and HMI (Figure 2C) spatial maps reveal concordance between high residual PPO activity and storage-induced browning, with R^2^ values of 0.71–0.87 (55). It should be noted that R^2^ is a measure of quantitative prediction accuracy, whereas classification accuracy is a measure of categorical discrimination. These metrics are not directly comparable, and acceptable thresholds depend on the specific application. Ma et al. (56) used enhanced PLS-DA with first-derivative processing to detect chilling injury in bananas stored at 7 °C versus 13 °C, achieving 100% classification accuracy 2 days before the onset of apparent symptoms. Real-world adaptability would be determined by validation under various postharvest handling conditions and crop seasons. HSI has been used to monitor pathogen-induced browning, in addition to physiological factors such as chilling injury. To track PPO activity under gray mold stress, Kai et al. (57) obtained SWIR spectra from 253 healthy and 257 infected cauliflower samples. The ability of HSI to non-destructively track pathogen-induced browning susceptibility was demonstrated by predictive models constructed using spectrophotometric PPO values as a reference. However, this strategy has two basic drawbacks. First, spectrophotometric PPO assays use excess substrate and ideal *in vitro* conditions to assess activity. These conditions do not reflect the behavior of *in situ* enzymes in damaged tissue. Second, actual browning development in the same samples was not used to validate spectral predictions of PPO activity. It remains unclear whether HSI detects PPO directly or merely records spectral changes associated with secondary tissue damage. Therefore, the reported R^2^ values should be interpreted with caution, as they reflect the ability of HSI to predict *in vitro* PPO activity rather than actual browning development in intact tissues. It is important to distinguish direct browning detection from detection of related tissue damage or stress. None of the cited studies directly quantified melanin formation. Bruise detection reflects tissue damage, PPO activity prediction reflects biochemical precursors, and chilling injury detection reflects physiological stress. These precursor signals indicate browning risk but do not confirm that browning has occurred. In conclusion, HSIs and MSIs effectively identify browning precursors, such as phenolic oxidation and membrane breakdown, days before symptoms become visible. Translation to commercial sorting lines, however, requires addressing cultivar variability, reducing computational requirements, and replacing browning validation of the target product with *in vitro* reference standards.

**FIGURE 2:**
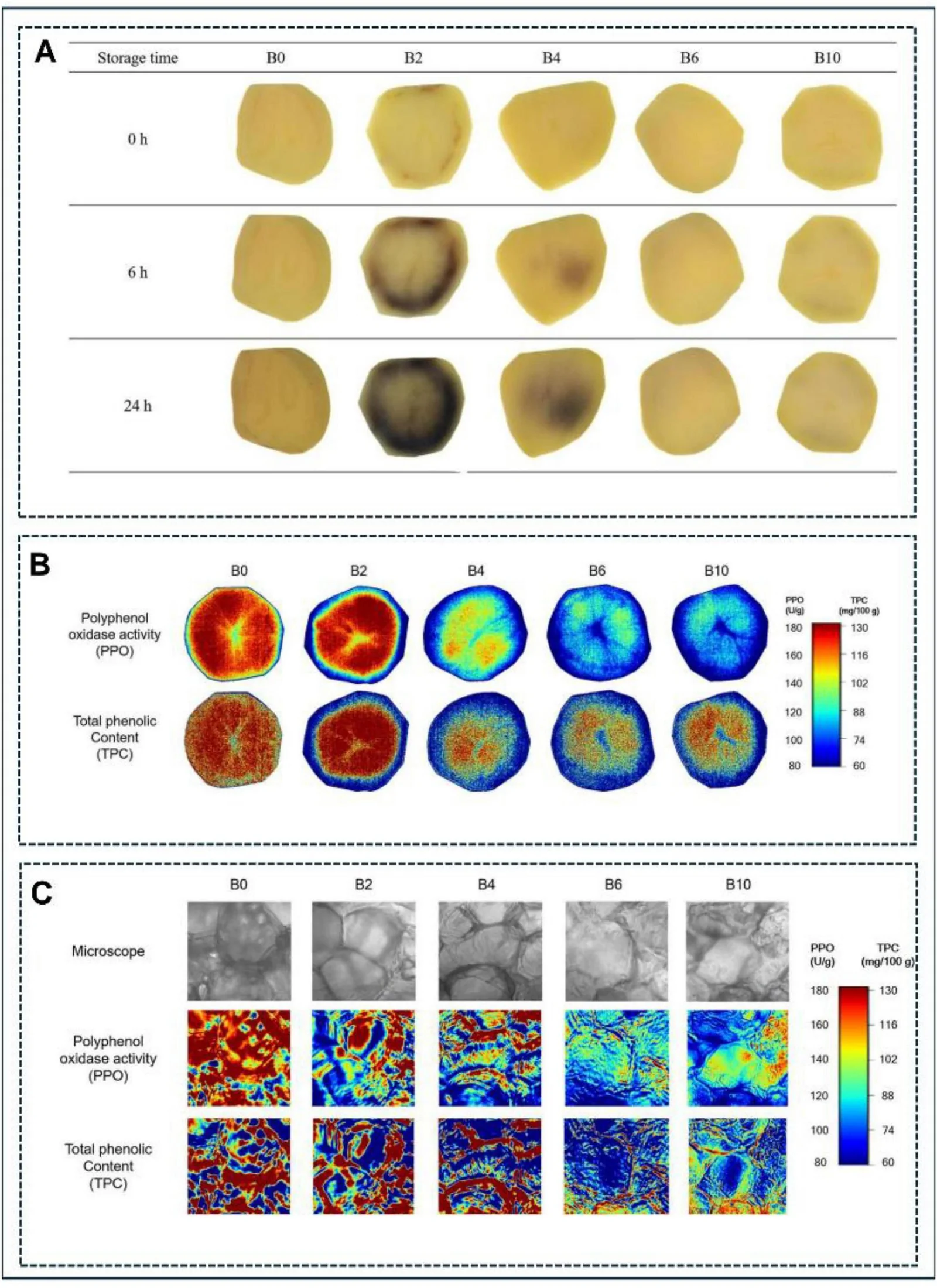
Hyperspectral imaging for non-destructive detection of enzymatic browning. **(A)** Red-green-blue (RGB) images of potato samples by blanching and storage times; **(B)** spatial distribution maps of PPO activity and total polyphenol content (TPC) in potato samples according to blanching time based on hyperspectral imaging. **(C)** Spatial distribution maps of PPO activity and total polyphenol content (TPC) in potato samples according to blanching time based on hyperspectral microscopic imaging. Reproduced from Park et al. (55) under the terms of Creative Commons Attribution (CC BY 4.0) License.

### 4.2 Chlorophyll fluorescence

Chlorophyll fluorescence analysis is a sensitive, non-destructive optical tool for detecting early-onset disorder symptoms in fresh-cut products because it allows tracking of alterations in chloroplast membrane integrity and photosynthetic efficiency before visible symptoms. Mechanical damage, chilling injury, controlled atmosphere (CA) disorders, and other stressors impair thylakoid membrane and photosystem II function. These perturbations are reflected in measurable fluorescence parameters, such as maximal fluorescence (Fm), minimal fluorescence (fo), and the quantum yield ratio Fv/Fm (57). Destruction of thylakoid membranes limits electron transfer between photosystem II and photosystem I. This defect increases Fo and reduces Fv/Fm, providing a quantitative measure of stress intensity before the onset of browning. Pliakoni et al. (58) demonstrated the predictive capacity of CF for brown stain in romaine lettuce, showing that Fv/Fm values were significantly lower under injurious controlled atmosphere conditions compared with non-injurious treatments, with symptoms appearing only after transfer to air. Kuang et al. (59) further validated CF as a senescence indicator by linking fluorescence parameters directly to the expression of chlorophyll degradation genes in flowering Chinese cabbage.

Despite its sensitivity, CF has important limitations that constrain its practical application for browning detection. The most significant limitation is that CF is exclusively applicable to green tissues containing functional chloroplasts. This excludes many products highly susceptible to enzymatic browning, including roots and tubers, inner flesh of pome and stone fruits, non-green fresh-cut products, and browning-susceptible storage organs. The mechanism underlying this limitation is intrinsic: CF measures photosynthetic efficiency, which requires an intact photosynthetic apparatus. Products that have been peeled, cut, or are naturally non-green lack functional chloroplasts in their edible tissues, rendering CF ineffective as a detection tool. Even in green tissues, the requirement for a 20–30-min dark adaptation period before measurement presents a practical challenge for high-throughput sorting lines, where product moves continuously at speeds exceeding 10 tons per hour (60). Furthermore, a decrease in Fv/Fm indicates photosynthetic stress but does not specifically prove that PPO-mediated browning will occur. Stress-induced declines in photosynthetic efficiency can result from factors unrelated to browning, including water stress, nutrient deficiency, temperature fluctuation, and pathogen infection (61). Therefore, CF should be interpreted as an indicator of browning risk or stress intensity rather than a direct measure of browning itself. In conclusion, CF provides useful early warning capabilities for green, chlorophyll-containing commodities such as unpeeled fruits, leafy vegetables, and green herbs. It is not appropriate for most browning-sensitive fresh-cut items, which are the main targets of anti-browning treatments, and its use is essentially limited to products with functional chloroplasts. In order to extend predictive capabilities to non-green items, future research should focus on integrating CF with other detection modalities.

### 4.3 Biosensors and electronic noses for volatile detection

E-nose technology can identify browning-related diseases by monitoring changes in VOC patterns that correspond with tissue deterioration. These metabolic changes are the molecular basis. Aldehydes, ketones, and alcohols are produced by membrane lipid peroxidation when tissue degrades because of mechanical damage, cold injury, or aging. Additionally, phenolic oxidation and microbial activity generate unique VOC profiles that E-nose sensors detect before visible browning occurs. Lai et al. (62) used an electronic nose and gas chromatography-mass spectrometry (GC-MS) to assess the extent of internal browning (IB) in pineapples. The classification accuracy of the radial basis function neural network (RBFNN) and multilayer perceptron neural network (MLPNN) models was 91.67% and 94.77%, respectively. Poeta et al. (63) measured the volatile profiles of Italian grapes stored at 2°C in a controlled atmosphere using an S3+ metal-oxide-semiconductor (MOS) nano-sensor. Fresh samples and those kept in optimal CA or air differed from grapes kept under extreme CA conditions, with E-nose results closely matching GC-MS data. By reducing browning and oxidation, both CA treatments improved visual quality. However, over time, they changed volatile content and increased respiration rates, which had a detrimental effect on scent. E-nose technology has two basic limitations. First, the E-nose simply sends a composite signal without chemical identification. Even when GC-MS and the E-nose are strongly linked to verify VOC pattern identification, GC-MS is still required to identify the volatile chemicals responsible for differentiation. Second, sensor drift occurs over time as baseline responses change due to sensor aging, contamination, or environmental variables. This limits long-term industrial deployment and requires regular recalibration (64). Additionally, commodity-to-commodity variation in VOC profiles poses a separate challenge, as models trained on one commodity may not transfer directly to another without recalibration. Table 1 compares detection technologies used for browning assessment.

**TABLE 1:** Comparison of detection technologies for browning assessment.

Technology	Principle	Primary endpoint measured	Sensitivity	Speed	Cost	Readiness level	Advantages	Limitations	References
Hyperspectral imaging	Spectral + spatial analysis	Biochemical precursors (phenolic oxidation, membrane breakdown, water redistribution)	High	Medium	High	TRL 7–8	Spatial distribution; early detection of precursors; detects PPO activity at ∼677 nm	Data processing intensive; cultivar-specific models	(54)
Multispectral imaging	Selected wavebands (R, B, NIR)	Biochemical precursors (surface changes)	Medium	High	Medium	TRL 9	Fast; suitable for in-line sorting; better spatial detail than colorimeters	Limited spectral information; requires image analysis	(139)
Chlorophyll fluorescence	Photosystem II efficiency	Physiological stress (photosynthetic efficiency; Fv/Fm)	Very high (pre-symptomatic)	Medium	Medium	TRL 6–7	Detects stress before visible symptoms; non-invasive through film bags	Only for green tissues; dark adaptation required; Fv/Fm reflects stress, not browning specifically	(140)
Electronic nose	Volatile organic compounds	Biochemical precursors (VOC profiles)	High	High	Medium	TRL 8–9	Detects internal disorders; correlates with sensory; non-invasive	Requires calibration per commodity; sensor drift; composite signal needs GC-MS confirmation	(62, 63)
Colorimetry (CIELab imaging)	Reflectance/CIELab color measurement	Direct browning (surface discoloration; BI, ΔBI, ΔE, ΔE_00_)	High	Very high	Low	TRL 9	Standardized; quantitative; portable; affordable high-throughput imaging now available	Surface measurement only; non-enzymatic browning may confound	(141)
VIS–NIR spectroscopy	Light absorption (350–1,150 nm)	Biochemical precursors (PPO/POD activity, multiple parameters)	Medium	High	Medium	TRL 8–9	Rapid; multiple parameters; automated enzyme activity estimation	Model development required; cultivar-specific calibration	(56, 142)

Definitions and criteria for qualitative ratings: Sensitivity; very high, detects pre-symptomatic changes; high, detects biochemical/spectral changes; medium, detects changes at later stages. Speed: very high, seconds; high, seconds to minutes; medium, minutes. Cost: low ≤ $5,000; medium = $5,000–$50,000; high ≥ $50,000. TRL values are indicative and based on commercial availability and published validation studies. Technologies measure different endpoints and should not be directly ranked.

## 5 Control strategies for enzymatic browning in fresh-cut fruits and vegetables

Treatment-induced reductions in PPO or POD activity are frequently reported as evidence of anti-browning efficacy. However, such reductions should not be interpreted as definitive proof that these enzymes are the sole or primary causal agents of browning. Treatments may reduce browning through multiple mechanisms, including membrane preservation, quinone trapping, antioxidant effects, and modulation of phenolic metabolism, which may occur independently of direct enzyme inhibition. Therefore, interpret enzyme activity data alongside browning index measurements, phenolic profiles, and membrane integrity assessments to establish causal relationships.

### 5.1 Traditional chemical inhibitors

#### 5.1.1 Sulfites

Sulfur dioxide (SO_2_) and its related compounds have been widely used for preventing enzymatic browning in perishable fruits and vegetables. Sulfites block both the enzymatic oxidation of phenols to reactive o-quinones and the subsequent non-enzymatic polymerization of these o-quinones into dark melanins, thereby accounting for their anti-browning effectiveness. Mechanistically, sulfites divert the quinone intermediates from the polymerization pathway by forming stable addition products with o-quinones (65). Despite this remarkable effectiveness, safety concerns have made the use of sulfites extremely contentious and increasingly restricted. Sulfite sensitivity can occur in susceptible individuals, particularly those with asthma, with symptoms ranging from urticaria and breathing difficulties to, in rare cases, anaphylaxis. Imsabai et al. (66) demonstrated that 1.5% sodium metabisulfite + ultrafine bubbles match the browning control of 3% SMS in coconuts, with UFBs synergistically mitigating microbial risks throughout the wet season. However, universal sulfite replacement remains unlikely because PPO isoforms respond unpredictably to natural extracts. In conclusion, because PPO isoforms respond inconsistently to plant extracts, inhibiting one PPO may activate another, thereby requiring product-specific anti-browning treatments, and a universal sulfite substitute is not possible.

Regulatory status of sulfites varies by jurisdiction and commodity. In the United States, the FDA prohibits the addition of sulfites to fresh fruits and vegetables intended to be consumed raw, except grapes, where sulfur dioxide is permitted for decay control with a residue tolerance of 10 μg SO_2_/g (67). Sulfites are allowed on some goods in the European Union by Regulation (EC) No 1333/2008. These goods include table grapes, fresh lychees, and blueberries (maximum 10 mg/kg), peeled potatoes (50 mg/kg), and vacuum-packed sweetcorn (100 mg/kg) (68). Section B.11.001.1 of the Food and Drug Regulations in Canada prohibits the sale of any fresh fruit or vegetable that is meant to be consumed raw, except grapes, if sulfites have been added (69). These regulatory distinctions reflect the balance between technological effectiveness and documented health risks. The prevalence of sulfite sensitivity among asthmatic patients has been estimated at 3.9% in a prospective challenge study, with steroid-dependent asthmatics being most at risk (70). A review of sulfite hypersensitivity indicated that 5%–10% of chronic asthmatics may be sulfite hypersensitive (71).

#### 5.1.2 Ascorbic acid and organic acid formulations

By directly chelating copper ions at the enzyme’s active site, forming stable complexes with quinone intermediates, and inducing pH-dependent conformational changes that reduce PPO catalytic activity, organic acids prevent enzymatic browning. The mechanism of copper chelation is particularly significant. PPO requires two copper atoms at the active site to bind oxygen and oxidize its substrate. The enzyme becomes inactive when its copper ions are sequestered by organic acids, independent of pH or substrate presence. But the efficacy of buffer systems does not often predict performance in real foods. Zhou et al. (72) revealed that organic acids are strongly dependent on PPO structure and the nature of the food matrix. This is because of restricted diffusion into tissue, antagonistic substrates, and buffering capacity, which result in differences. An inhibitor that is active *in vitro* might not act under complex conditions within damaged plant tissue, where pectin, cellulose, and competing phenolics vary in diffusion and binding. Building on this insight into matrix dependence, derivatives of organic acids have since been studied to identify structural elements that contribute to inhibitory activity. The most effective inhibitors in the experiment on cinnamic acid and its derivatives against PPO, between pH 6.8 and 5.0, were found to be 4-carboxycinnamic acid, cinnamic acid, and methyl cinnamate (73). Acidic pH amplified the effect of the inhibitor, whereas the IC50 and Ki values were reduced, respectively. The relationship between form and action is further revealed: the unsaturated side chain promotes the maximum orientation of copper coordination, and the carboxyl group at the 4-position promotes binding at the PPO active site.

While cinnamic acid derivatives show promise in solution-based assays, the physiologically relevant form of PPO in intact tissues is membrane-bound rather than soluble. This distinction has notable implications for inhibitor screening. Ascorbic acid, cinnamic acid, β-cyclodextrin, and L-cysteine were tested against membrane-bound (mPPO) and soluble (sPPO) PPO in peach fruit. Ascorbic acid inhibited sPPO by 95.42% at a dose of 1 mM, whereas it only inhibited mPPO by 65.60% (74). There are significant implications to this difference. The physiologically significant form of PPO in intact tissues where browning takes place is membrane-bound PPO. Soluble enzyme preparations are widely used in inhibitor screening studies due to their ease of extraction and testing, and they remain valuable for mechanistic screening of inhibitor-enzyme interactions. However, because sPPO is not the physiologically dominant form in intact tissues, results obtained with sPPO may not fully predict inhibitor performance in intact food matrices, as demonstrated for ascorbic acid with peach fruit PPO. Reported IC50 values for ascorbic acid and related compounds may not fully reflect actual browning control, as suggested by the difference observed between sPPO and mPPO in this study. It should be noted that this comparison was conducted using ascorbic acid and peach fruit PPO specifically, and the extent to which this difference applies to other inhibitors, PPO sources, and food matrices remains to be determined through broader comparative studies. In conclusion, ascorbic acid is still effective, but only against soluble PPO, and 4-carboxycinnamic acid exhibits promise based on structure-activity connections. But translation requires changing from buffer systems to real food matrices and from soluble enzyme screening to membrane-bound PPO testing.

### 5.2 Traditional physical methods

The most common postharvest preservation method for perishable fruits and vegetables is cold storage, which mainly inhibits enzymatic browning by lowering respiration rates and delaying senescence-related membrane breakdown (75). The temperature-dependent decrease in PPO and POD catalytic activity, along with the preservation of membrane integrity, which maintains subcellular compartmentalization between enzymes and phenolic substrates, are the mechanisms underlying the effectiveness of cold storage (31). However, browning may be paradoxically accelerated by extended cold storage followed by a sudden change to room temperature during supply chain transitions. Extended cold storage enhanced litchi browning by 41.9% compared to 10 days of storage, with 9%–28% decreases in antioxidant enzymes and 1.2–3.6 increases in POD. This established ideal limits of 10 days + 6 h and 20 days + 12 h, beyond which permanent deterioration occurred (76). Thermal blanching reduces PPO and POD activity, primarily through heat-induced denaturation of their tertiary structure. However, the contribution of enzyme inactivation to overall browning reduction may vary depending on the commodity and treatment conditions. While steam produced 97.1% POD inactivation and 52.8% PPO reduction and, with 90 s of steam, ideally preserved phenolics and cyanidin-3-O-glycosides, hot water blanching inactivated 89.6% PPO and 92.1% POD in mangosteen but degraded anthocyanins (77). Similarly, Fresh-cut yams were cured at a high temperature, which decreased the formation of phenolics and lignin while suppressing PPO, POD, PAL, C4H, and 4CL activities. While delayed browning requires PAL suppression through modest heat shock, this focuses on the acute browning phase (31). In conclusion, despite its effectiveness, traditional thermal blanching degrades quality by causing nutrient loss, softening of texture, and color changes. This has led to a shift toward new non-thermal treatments, which will be covered in the sections that follow.

### 5.3 Atmospheric modification

The two main approaches for controlling enzymatic browning by adjusting the quantities of carbon dioxide and oxygen around fresh-cut products are modified atmosphere packaging (MAP) and controlled atmosphere storage (CA). Zhang et al. (78) applied a multi-omics approach to atemoya fruit using MAP with microperforated packaging. Through simultaneous effects on ascorbate metabolism and cell wall integrity, the treatment reduced the browning index and increased shelf life by 12 days. However, because it is multi-omics research, it identifies associations rather than causation. In the case of a change in the MAP-to-CA comparison, the treatments were evaluated on fresh-cut Chinese water chestnuts. CO_2_ was the most effective, eliminating 94.39% of browning within 8 days and simultaneously inhibiting enzymatic oxidation and phenolic formation (79). This 94.39% reduction, although the deductive framework was based on enzyme assays, represents a high level of efficacy compared with other atmospheric modification studies. In the case of fresh-cut eggplant, Lemos et al. (80) combined active MAP with light, heat, and citric acid, and stored their produce for 10 days, maintaining PPO activity at 2.8 U mg-1 protein, antioxidant capacity at 70%, and ascorbic acid pressure at 64%. This hurdle technique, unlike the one-factor MAP planned by Zhang or the CA-only method by Liu, demonstrates that a change in the atmosphere may not be sufficient. Rather, MAP with a little heat and acid is used successfully in cases of MAP failure. But the synergistic mechanism has not been defined in the study, nor does it isolate the effects of heat, citric acid, or MAP. In the study of pears by Leisso et al. (81), it can be observed that although rapid cooling after harvest, followed by air storage or delayed CA establishment, reduced the increase in browning, high CO_2_ and immediate CA establishment increased the occurrence of internal browning disorder. This directly contradicts the CO_2_-benefit narrative of Liu and Lemos. Pears may be CO_2_-sensitive, whereas water chestnuts and eggplant either tolerate or benefit from increased CO_2_. This variation is probably due to product-specific physiology. The other studies don’t address this important point: that increasing CO_2_ isn’t always beneficial. McTavish et al. (82) examined CO_2_-induced interior browning in apples stored at ultra-low pO_2_ and elevated pCO_2_. Acylated steryl glycosides, free fatty acids, and sphingolipids are lipid biomarkers that increase before the onset of symptoms, whereas steryl esters and certain amino acids decrease with risk. This work identifies pre-symptomatic biochemical markers of fruit damage, enabling prediction rather than post hoc identification. Nevertheless, translation requires quick, non-destructive detection of these lipids, which is currently unavailable. In conclusion, atmosphere modification reduces browning by up to 94% through O_2_/CO_2_ control. However, effectiveness varies by product, and design challenges make it impossible to isolate the role of MAP/CA.

### 5.4 Edible coatings and films

Edible coatings prevent enzymatic browning by acting as semi-permeable barriers that limit oxygen passage and prevent PPO from accessing a necessary co-substrate. They also act as carriers for anti-browning chemicals that penetrate the cut surface. Orhotohwo et al. (83) extended this carrier concept to waste valorization by using spent coffee ground extract (SCGE) to optimize chitosan-alginate films. Over 10 days at 4 °C, the ideal formulation reduced moisture loss by 37.5% and scavenged 87.52% of DPPH in fresh-cut kiwis. The anti-browning mechanism was inferred rather than confirmed because this study did not directly test PPO activity or quinone production, but it did provide mechanical and hydrophobicity data, including 6.33 MPa and a contact angle of 78.31°. Yin et al. (84) demonstrated that a 4% peach gum coating on “Guiwei” litchi inhibited POD activity and decreased phenolic formation at room temperature, shifting from polysaccharide-based to gum-based coatings. Unlike the trial on refrigerated kiwis, this study showed effectiveness in ambient conditions, which are more difficult to prevent browning. Magri et al. (85) used citric and oxalic acids in sodium alginate-carboxymethyl cellulose coatings on fresh-cut pears, switching from single-layer to layer-by-layer systems. Over 10 days at 2 °C, this multi-layer strategy reduced protein degradation and increased ROS-detoxifying enzyme activity. This study offers a thorough mechanistic understanding by linking coating applications to specific enzyme pathways rather than merely reporting color or moisture results. In conclusion, edible coatings prevent browning via barrier and carrier synergy, but inconsistent conditions and the lack of direct comparisons of inhibitors prevent ranking of which coating works best.

### 5.5 Thiol-rich compounds as quinone trapping agents

Another mechanism for controlling browning is substrate complexation and quinone trapping. These methods target the reactants or products of enzymatic oxidation rather than the enzyme itself. By forming inclusion complexes with phenolic compounds, cyclodextrins act as substrate-complexation agents, delaying their oxidation to quinones (86). The aromatic ring of phenolics is physically blocked from accessing PPO’s active site by the hydrophobic cavity of cyclodextrins. Despite their demonstrated effectiveness in model systems, the commercial application of cyclodextrins remains limited. They are not viable for most fresh-cut applications due to high production costs and the need for high molar ratios to achieve considerable browning inhibition. A different approach binds quinones after they are created, blocking their polymerization into melanins, whereas substrate complexation inhibits quinone production.

A deeper mechanistic understanding of thiol-quinone interactions has been made possible by the most recent advances. Cysteine and glutathione prevent browning by forming colorless thiol-quinone adducts via nucleophilic addition rather than by permanently blocking PPO. The antibrowning effect of the resultant additives comes only from sequestering quinone intermediates before polymerization into melanins (87). Wang et al. (88) confirmed phenolic-dependent adduct stability by demonstrating that pyrimidine-dithiol (PDT) provides 13-fold stronger tyrosinase inhibition than kojic acid via bidentate copper chelation. Complementing these findings, the differences in reactivity between aliphatic and aromatic thiols have been systematically characterized. Aromatic thiols consume quinones quickly because of their greater nucleophilicity, while aliphatic thiols take about 15 min. Rapid trapping reduces polymerization, facilitating control of browning at lower concentrations (89).

Building on these insights into thiol-quinone chemistry, Ethyl 3-mercaptopropionate (EMP) was found by Sui et al. (8) to be a strong quinone-trapping agent with nanomolar sensitivity against pure potato PPO. Through competitive inhibition, EMP attaches itself to copper-coordinating histidine residues at the active site. Since EMP is found naturally in Concord grapes and ripened cheeses, the European Union and JECFA have certified it as safe. However, quick commercial use is restricted by two challenges. According to Mirza et al. (90), concentrations above 100 μL/L may result in a significant sulfurous odor that may influence consumer acceptance. Second, the membrane-bound versus soluble issue discussed in the preceding section was raised when the nanomolar concentration was determined using soluble PPO preparations. Although there is no systematic commercial comparison, it remains unclear whether thiols act via direct copper chelation or quinone trapping. The difference is significant for high-PPO vs. less-processed products. In conclusion, recent research has established thiol structure-activity relationships, thereby demonstrating the mechanistic validity of quinone trapping. Cost reduction, sensory threshold assessment, validation of the phenolic profile, and significant validation against membrane-bound PPO are all required for commercial applications.

### 5.6 Emerging physical technologies

#### 5.6.1 Non-thermal technologies

##### 5.6.1.1 Pulsed electric fields (PEF)

A promising non-thermal method for regulating enzymatic browning through electroporation-induced structural changes of PPO and other oxidative enzymes is pulsed electric field (PEF) technology. Rashvand et al. (52) demonstrated that PEF treatment enhances cell membrane electroporation, thereby facilitating mass transfer while preserving fruit and vegetable quality attributes, including color, phytochemicals, and antioxidant capacity. The speed of treatment applications enables significant savings in processing time and energy consumption compared with conventional methods. Nevertheless, the study has identified a few drawbacks, such as potentially high energy costs and partial inactivation of some hardy microbial strains, which could lead to regeneration during storage. Evrendilek and Özkan (12) studied PEF processing of fresh grape, orange, and tomato juices at 17.2–24.1 kV/cm with treatment times of 130.0–1040.5 μs, moving on from review to juice applications. They demonstrated that prolonged treatment periods resulted in over 95% inactivation of the enzymes LOX, pectin methyl esterase (PME), and PPO, without compromising qualitative properties. Different parameters are needed when applying PEF to solid produce. PEF was used by Katsouli et al. (91) before the osmotic dehydration of fresh-cut potatoes. The dehydration efficiency index for PEF-treated samples reached 2.10, compared with untreated controls, indicating that PEF pretreatment, combined with anti-browning chemicals in the osmotic solution, greatly enhanced color retention. However, PEF’s contribution was not distinguished from that of the co-applied inhibitors in the study. A study on jabuticaba fruits indicated a different use. The study evaluated PEF pretreatment before freeze-thaw cycles and found that 0.75 kV/cm best protected fruit quality, reducing the freezing phase-transition zone time by 18.3% compared with untreated samples (92). In conclusion, PEF reduces PPO activity through multiple mechanisms that may include electroporation, electric-field-induced structural changes, and thermal effects depending on treatment intensity. However, studies often fail to isolate the contribution of PEF from co-applied inhibitors, directly measure PPO activity, or compare efficacy against conventional ascorbic acid dips. The relative contribution of these mechanisms likely varies with product, enzyme source, and treatment conditions, leaving the industry without clear guidance on cost-benefit.

##### 5.6.1.2 Cold plasma

Three approaches are used by cold plasma (CP) to prevent enzymatic browning: structural denaturation of enzymes, direct deactivation of PPO via reactive species, and regulation of oxidative stress responses. CP produces RONS that chemically alter PPO’s active site, representing a fundamentally different inactivation mechanism from PEF, which depends on electroporation (93). The mechanisms by which CP generates its inhibitory effects are schematically depicted in Figure 3A. The plasma is generated when a gas is exposed to a high-voltage electromagnetic field, producing a state consisting of free radicals, excited atoms, and charged particles. This process yields a rich broth of reactive species, including oxygen and nitrogen species (ROS and RNS). As illustrated in Figure 3B, these reactive species are the primary agents responsible for the subsequent inactivation of food enzymes (94). Du et al. (13) achieved 60% PPO inhibition over 10 days at 4 °C by optimizing CP with 10% jujube honey on fresh-cut apples. Nevertheless, it is not addressed whether the 40% residual activity resulting from 60% inhibition is adequate for commercial use. Shirazi et al. (95) applied a higher voltage but a shorter duration to patulin-contaminated apples, using 23 kV for 2.5–10 min. On days 1, 7, and 14, PPO activity dropped by 12%, 47%, and 28.5%, respectively. Although the 28.5% inhibition by day 14 is significantly less than Du’s 60%, a direct comparison is complicated by the various products and CP systems. When Zheng et al. (96) applied high-voltage CP to fresh-cut Hami melons, they were able to achieve 1.06 log microbial reduction and substantial PPO/POD inhibition over a period of 8 days at 4 °C. This is one of the few studies to report both microbiological and enzymatic findings relevant to fresh-cut safety. Cao et al. (97) mechanistically demonstrated that CP fumigation inhibited Botrytis cinerea in apples through metacaspase-dependent apoptosis, a ROS burst, and mitochondrial calcium elevation. Although this clarifies the antifungal mechanism of CP, neither PPO activity nor browning was explicitly measured in the study. Its inclusion here depends on the idea that pathogen-induced browning is indirectly prevented by fungal suppression. CP reduces PPO activity via RONS-mediated structural modification. However, the relative contribution of direct enzyme inactivation versus other mechanisms to overall browning reduction requires further investigation.

**FIGURE 3:**
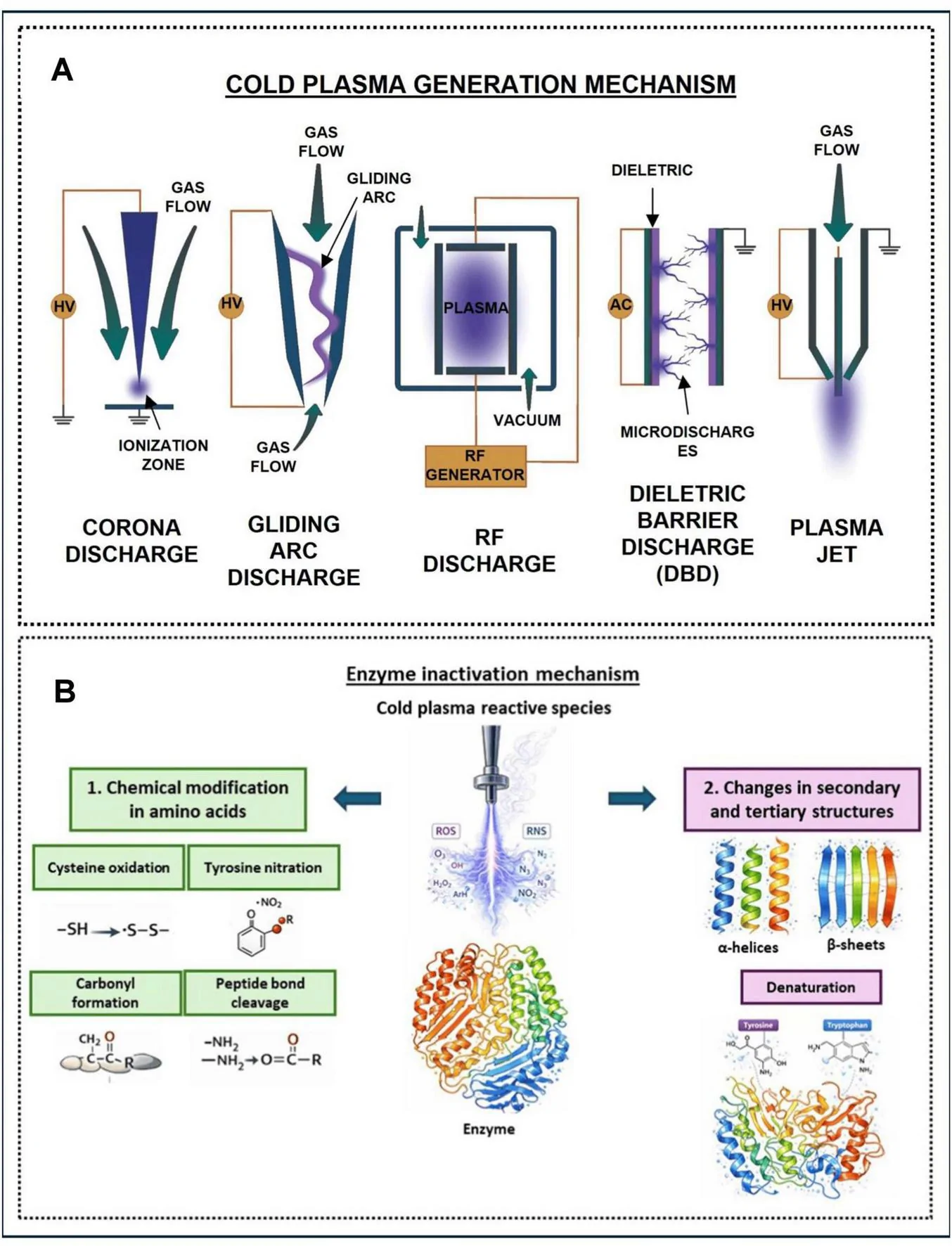
**(A)** Schematic diagram of the mechanisms for generating cold plasma; **(B)** enzyme inactivation mechanism caused by reactive species generated by cold plasma. Reproduced from Rigolon et al. (94) under the terms of Creative Commons Attribution (CC BY 4.0) License.

##### 5.6.1.3 High-pressure processing

One method of inactivating PPO is high-pressure processing (HPP), which disrupts the tertiary structure and dissociates oligomeric enzymes. But it is highly matrix-dependent and often requires a combination with other treatments. Tian et al. (98) provide an example of the limitations of HPP alone: high hydrostatic pressure alone decreased PPO activity by just 9.0%. Nevertheless, the synergistic combination resulted in over 95 percent inactivation in combination with ferulic acid (FA) and epigallocatechin gallate (EGCG). This 10-fold improvement demonstrates that HPP can be a more effective delivery modulator than an independent inhibitor. Conversely, Reddy et al. (99) tested HPP on soft coconut water. They attained a much lower inactivation rate with phenolics and vitamin C in good condition. This experiment established that high pressure alone was insufficient to maintain high-PPO products in the absence of phenolic co-inhibitors.

Ning et al. (100) applied this combination approach to blueberry juice and found that using 2 g/L gallic acid with HPP reduced PPO relative activity to 28.13% and reduced anthocyanin degradation to 13.42% after 20 days at 4 °C, in comparison to 50.78 PPO relative activity and 62.27 anthocyanin degradation. From a mechanistic perspective, this leads Tian to conclude that co-treatments with phenolic compounds are necessary. Nonetheless, Ning had a residual activity of 28%, compared with Tian’s 5%, which means that the phenolic structure and pressure conditions determine the ultimate efficacy. Sulaiman and Silva (101) did a comparison of HPP-thermal, thermosonication, and thermal treatment alone on purees of strawberries, apples, and pears, yielding the most important conclusion. In contrast to thermal treatment alone at 71 °C, HPP-thermal did not improve PPO inactivation, despite the narrative of synergy. Of all the technologies, Pear PPO showed the greatest resistance. This work raises concerns about the notion that HPP always outperforms thermal processing, as any pressure benefit may be offset by product-specific enzyme resistance. In summary, HPP alone achieves limited PPO inactivation in many products, but its efficacy depends on the product matrix, enzyme source, and treatment intensity. Combining HPP with phenolic co-inhibitors can substantially improve inactivation. However, HPP-thermal is not always superior to thermal treatment alone, as observed for pear PPO. Therefore, HPP should not be considered universally effective or ineffective; its application must be optimized for each specific product and enzyme system.

##### 5.6.1.4 Ultrasonication

Polyphenol oxidase becomes inactivated by the cavitation effect of ultrasound (US), resulting in structural denaturation: microbubble implosion produces high temperatures, free radicals, and localized shear forces that disrupt tertiary structure and hydrogen bonding (102). Fu et al. (103) showed synergistic inhibition of ultrasonography with oxyresveratrol in fresh-cut potatoes, showing only a 19% net PPO decrease after 96 h. This intermediate effectiveness casts doubt on the efficacy. Mechanistically, a decrease in the β-helix and random coil content ruled out secondary damage, and a 341–335 nm spectral shift and fluorescence quenching affirmed tertiary disruption. Advancing mechanistic depth, Jin et al. (104) subjected *Agaricus bispanus* to dual-frequency US in the presence of electrolytic water (DFU-EW). Treatment increased random coil and β-sheet while decreasing β-helix and β-turns. Conformational collapse was confirmed by a decrease in surface hydrophobicity and fluorescence intensity. PPO activity stabilized at 60% residual over 7 days, while X-ray diffraction revealed a 7% increase in crystallinity, outperforming Fu’s single-frequency method and providing detailed structural insight.

In juice matrices, Juliato et al. (105) subjected Jabuticaba juice to high-intensity US. Notably, HIUS inactivated PPO, POD, and PME while increasing cyanidin-3-O-glucoside by 40% and gallic acid by 55%. However, cross-study comparison is not possible due to the lack of defined PPO inactivation percentages. In another study, Wang et al. (106) achieved PPO and POD activities at 72%–84% and 57%–60% of the control, respectively, using UV-US atomization coating on fresh-cut apples, with a 3.8–4.0 log CFU/g pathogen reduction. Although the challenge structure offers both rapid UV disinfection and long-term ascorbic acid protection, the independent effect of US remains unclear. Franco et al. (107) used UV-C, Microwave (MW), and US to treat banana puree. US reduced PPO activity and the browning index, while retaining 75% of the phenolic content. There was an increase in POD activity, which is paradoxical, and it typifies matrix-dependent enzyme sensitivity. This product-specific aberration did not occur in experiments on apples or mushrooms. In conclusion, dual-frequency electrolytic water systems have the most mechanistic rigor but have not been commercially validated. US reduces PPO activity via cavitation-induced conformational changes. However, the causal relationship between enzyme inactivation and browning reduction is not fully established, as US may also affect membrane integrity and phenolic metabolism.

##### 5.6.1.5 UV irradiation

A successful non-thermal method for regulating enzymatic browning is ultraviolet (UV) irradiation, particularly UV-C and UV-B, which both directly inactivate enzymes and strongly stimulate plant defensive responses (108). Optimized UV-C dosages reduced PPO activity and maintained color characteristics in fresh-cut Chinese yams throughout storage. However, replication and comparison were limited by the study’s failure to define UV intensity or exposure length (109). Moving from single- to comparative wavelength analysis, UVA, UVB, and UVC were systematically assessed on fresh-cut spinach stored at 4 °C for 10 days. The UV-B treatment resulted in the least amount of weight loss and the highest dry matter content. Importantly, exposure to UV-C increased electrical conductivity, suggesting a greater potential for oxidative damage and membrane ion leakage. This finding has important implications, as membrane integrity may be compromised by UV-C’s greater PPO inactivation (110). Shifting from post-harvest to pre-harvest application, Zhang et al. (111) studied UV-B treatment on Mesembryanthemum crystallinum before harvest. While low-intensity UV-B activated the enzymatic and non-enzymatic antioxidant machinery to counteract ROS buildup, high-intensity UV-B caused declines in soluble protein and sugar levels amid increased oxidative stress. However, because the study examined oxidative stress markers rather than PPO activity or browning directly, the translatability of pre-harvest UV-B to fresh-cut browning control has not been validated. In conclusion, UV light regulates browning through direct PPO photoinactivation and stimulation of defensive responses; however, wavelength-dependent efficacy varies, and the mechanisms underlying commodity-dependent phenolic responses remain unknown.

#### 5.6.2 Electrothermal technologies

##### 5.6.2.1 Ohmic heating

By passing an electric current directly through food, ohmic heating (OH) produces rapid, volumetric heat that inactivates PPO more effectively than traditional thermal processing, since heat is generated internally rather than conveyed from a surface (112). Mari et al. (113) optimized OH for fresh-cut potatoes, successfully inactivating the enzyme and obtaining an overall acceptability score of 8.5. However, because the study did not report precise PPO inactivation percentages, it could not quantitatively compare its results with those of other studies. Barati et al. (114) examined PPO inactivation kinetics in carrot juice during OH shifting from solid produce to liquid systems. OH is preferable mechanistically because it generates heat instantly and uniformly, avoiding the temperature gradients that inhibit conventional heating. However, it is impossible to compare with other studies that use different electrode configurations, since constant voltage is used rather than electric field intensity. Kumar and Srivastava, (112) extended continuous processing by applying continuous OH to pineapple juice, and the maximum inactivation was 68.2% for PPO and 82.2% for POD. Notably, this 68.2% PPO inactivation is significantly lower than the levels typically achieved by conventional thermal processing under comparable time-temperature combinations, suggesting that processing speed, rather than superior enzyme inactivation, may be OH’s advantage. The most effective results were obtained by Chimsook and Assawarachan (115), who used the Box-Behnken design to optimize OH pasteurization for passion fruit juice. PPO and POD were inactivated to residual activities of 12.6% and 8.07%, respectively, under ideal conditions. Unlike the above studies, this work provides comprehensive data collection, including a direct comparison with traditional processing. In summary, Ohmic heating inactivates PPO via volumetric heating; however, cross-study comparisons and optimization are limited by inconsistent parameter reporting and a lack of mechanistic research on non-thermal electric-field effects.

##### 5.6.2.2 Microwave heating

Microwave treatment has also emerged as a successful thermal method for reducing enzymatic browning in fresh-cut products, compared with conventional blanching, due to its rapid, volumetric heating, which inactivates PPO and POD and better preserves quality properties than other blanching methods (116). Fang et al. (117) compared microwave blanching (MWB) and steam blanching of apple slices. MWB maintained phenolic levels and inhibited browning more effectively. MWB-induced microporous channels within cellular tissue were identified through microstructural analysis. These channels accelerate moisture diffusion and reduce drying time and specific energy consumption. Mechanistically, this pore formation provides an additional benefit that is not present in traditional blanching: MW treatment restructures tissue for improved downstream processing, in addition to inactivating enzymes. Shifting from a solid to a liquid matrix, Dayma et al. (116) evaluated the effects of MW heating on sugarcane juice. MW surpassed ultrasonic and combination treatments in maintaining physicochemical and phytochemical qualities while successfully lowering spoilage enzymes. PPO and POD were inactivated. However, quantitative comparison with other juice research was not possible because the study did not give particular PPO inactivation percentages. In contrast to conventional thermal treatment, which required 95 °C for 5 min to achieve equivalent performance, Dhar and Chakraborty (118) showed that continuous MW processing of bael beverage produced ≥ 90% PPO inactivation at 90 °C end temperature. Additionally, MW exhibited greater antioxidant activity and higher overall phenolic retention. because MW’s volumetric heating reduces thermal over-exposure at the product surface, hence minimizing collateral nutrient degradation, this 5 °C temperature difference is mechanistically relevant. Fatchurrahman et al. (119) reported that pre-cutting MW treatment of entire artichokes is more effective than post-cutting application. This is probably because enzyme inactivation within the whole tissue prevents phenolic leaching. In conclusion, Microporous channel building and pre-cutting application tactics are secondary benefits of microwave treatment, which provides more than 90% PPO inactivation at temperatures 5 °C below those achieved by traditional volumetric heating. However, systematic optimization is restricted by inconsistent parameter reporting.

#### 5.6.3 Combined/hurdle technologies

Many new technologies work better when used in conjunction with other treatments, producing synergistic results that are difficult to achieve with just one treatment. Notable examples include HPP in combination with phenolic co-inhibitors, which significantly improves PPO inactivation when compared to HPP alone; PEF in combination with osmotic dehydration and anti-browning chemicals, which improves color retention; CP in combination with jujube honey, which achieves 60% PPO inhibition; and US in combination with oxyresveratrol or UV-US atomization coating, which offers both disinfection and anti-browning protection. These combined approaches are discussed within their respective subsections and summarized in Table 2. The rationale for combined approaches is to achieve synergistic effects while reducing treatment intensity, minimizing quality degradation, and preserving product quality. However, most studies do not isolate the contribution of individual components; optimal concentration ratios and treatment sequences have not been systematically established, and efficacy is highly product-dependent.

**TABLE 2:** Efficacy of novel control strategies across different fresh-cut products.

Treatment	Product	Optimal conditions	Enzyme activity response	Browning response	Quality/ microbial response	Shelf-life outcome	Evidence type	Sensory	Microbial	References
Chemical inhibitors										
Sodium metabisulfite + ultrafine bubbles	Coconut	Dip, wet season	–	Matched 3% SMS control	Microbial risk mitigated	–	FM	–	✓	(66)
Ascorbic acid	Peach (sPPO)	Dip	95.42% sPPO inhibition	–	–	–	sPPO	–	–	(74)
4-carboxycinnamic acid	PPO assay	pH 5.0–6.8	Most potent cinnamic derivative	–	–	–	PE	–	–	(73)
EMP	Fresh-cut potato	Dip	>90% PPO inhibition	–	–	6–8 days (storage duration)	FM	–	–	(143)
Carbamate	Chokeberry PPO	*In vitro*	Most potent carbamate	–	–	–	PE	–	–	(144)
L-pyroglutamic acid	Fresh-cut potato	Metabolomics-derived	PPO inhibition	–	Antimicrobial	–	FM	–	✓	(145)
Tropolone/thujaplicin	Recombinant pea PPO	*In vitro*	Most potent among tested	–	–	–	PE	–	–	(146)
P-coumaric acid	Apple	Competitive inhibition	sPPO more susceptible than mPPO	–	–	–	sPPO/mPPO	–	–	(147)
Kojic acid	Apple	Competitive inhibition	sPPO more susceptible than mPPO	–	–	–	sPPO/mPPO	–	–	
Chlorogenic acid	Apple	Mixed inhibition	Reversible inhibition	–	–	–	sPPO/mPPO	–	–	
Traditional physical										
Extended cold storage	Litchi	10 days + 6 h	–	41.9% increase if exceeded	–	10 days (storage duration)	ST	–	–	(76)
Steam blanching	Mangosteen	90 sec	52.8% PPO / 97.1% POD inactivation	-	Anthocyanins preserved	-	FM	-	-	
Hot water blanching	Mangosteen	–	89.6% PPO/92.1% POD inactivation	–	Anthocyanins degraded	–	FM	–	–	(77)
High-temperature curing	Fresh-cut yam	Heat shock	Suppressed PPO, POD, PAL, C4H, 4CL	–	–	–	FM	–	–	(31)
Atmosphere modification										
MAP	Atemoya fruit	MAP packaging	–	Reduced browning index	–	12 days (storage duration)	ST	–	–	(78)
CA	Fresh-cut Chinese water chestnut	8 days storage	–	94.39% browning reduction	–	8 days (storage duration)	ST	–	–	(79)
Active MAP + mild heat + citric acid	Fresh-cut eggplant	10 days at 4 °C	PPO constant at 2.8 U⋅mg^–^^1^	–	–	10 days (storage duration)	ST	–	–	(80)
High CO_2_ + rapid CA	Pear	Postharvest	–	Increased internal browning	–	Negative effect	ST	–	–	(81)
Edible coatings										
Chitosan-alginate + SCGE	Fresh-cut kiwi	10 days at 4 °C	–	–	87.52% DPPH scavenging	10 days (storage duration)	FM	–	–	(83)
Peach gum	“Guiwei” litchi	Ambient temperature	POD inhibited	Browning index suppressed	–	–	FM	–	–	(84)
Sodium alginate-CMC + citric acids	Fresh-cut pear	10 days at 2 °C	–	–	Reduced protein degradation + ROS detox	10 days (storage duration)	ST	–	–	(85)
Chitosan + polygonatum essential oil	Fuji apple	120 days storage	PPO inhibition	–	Decay rate 9.18%	120 days (storage duration)	ST	–	–	(148)
Rambutan peel extract + chitosan	Green oak lettuce	Encapsulation, 4 °C	–	–	Ellagic acid binding −6.75 kcal/mol	–	MS	–	–	(149)
Quinone trappers										
β-cyclodextrin	Model systems	High molar ratio	–	Effective but costly	–	–	MS	–	–	(86)
Cysteine/glutathione	Various	Nucleophilic addition	–	Colorless adduct formation	–	–	MS	–	–	(87)
EMP	Fresh-cut potato	Dip	Nanomolar sensitivity	–	–	6–8 days (storage duration)	FM	–	–	(8)
Emerging physical										
PEF	Grape, orange, tomato juices	130–1,040 μs	>95% PPO inactivation	–	–	–	FM	–	–	(12)
PEF	Jabuticaba	Pre-freeze-thaw	–	–	Reduced freezing time by 18.3%	Quality protected	FM	–	–	(92)
Cold plasma + 10% jujube honey	Fresh-cut apple	10 days at 4 °C	60% PPO inhibition	–	–	10 days (storage duration)	ST	–	–	(13)
Cold plasma	Patulin-contaminated apple	14 days	28.5% PPO inhibition	–	–	–	ST	–	–	(95)
High-voltage cold plasma	Fresh-cut Hami melon	8 days at 4 °C	Substantial PPO/POD inhibition	–	1.06 log microbial reduction	8 days (storage duration)	ST	–	✓	(96)
HPP alone	Various	Pressure only	9.0% PPO inactivation	–	–	–	MS	–	–	
HPP + ferulic acid + EGCG	PPO assay	Synergistic	>95% inactivation	–	–	–	PE	–	–	(98)
HPP + gallic acid	Blueberry juice	20 days at 4 °C	28.13% residual PPO activity	–	–	20 days (storage duration)	ST	–	–	(100)
Ohmic heating	Passion fruit juice	Box-Behnken design	12.6% residual PPO/8.07% POD	–	–	–	FM	–	–	(115)
Continuous ohmic heating	Pineapple juice	–	68.2% PPO / 82.2% POD inactivation	–	–	–	FM	–	–	(112)
Dual-frequency ultrasound + electrolytic water	*Agaricus bisporus*	7 days	60% residual PPO activity	–	–	7 days (storage duration)	ST	–	–	(104)
Ultrasound + oxyresveratrol	Fresh-cut potato	96 h	19% net PPO decrease	–	–	–	FM	–	–	(150)
Microwave blanching	Apple slices	–	–	Better than steam blanching	Microporous channels	Reduced drying time	FM	–	–	(117)
Continuous microwave	Bael beverage	90 °C end temp	≥90% PPO inactivation	–	–	–	FM	–	–	(118)
UV-C	Fresh-cut Chinese yam	–	–	–	–	Extended storage	ST	–	–	(109)
UV-B	*Mesem bryanthemum crystallinum*	Pre-harvest	–	–	Activated antioxidant machinery	–	FM	–	–	(111)
Natural and bio-based extracts										
Pomegranate peel extract	Potato, mushroom, apple	4 °C	IC_50_: 0.73–0.86 mg/mL	–	–	3–7 days (storage duration)	PE	–	–	(11)
Fermented avocado seed extract	Avocado, banana, apple	0.5%–1.0% v/v	Total PPO inhibition (competitive)	–	–	Significant delay	FM	–	–	(120)
Galla rhois water extract	Apple juice	250–1,000 μg/mL	–	Outperformed vitamin C	–	16 days (storage duration)	FM	–	–	(121)
Sugarcane straw + yeast mannan	Fresh-cut apple	5 g/L mannan	More PPO inhibition	Less color loss	–	–	FM	–	–	(122)
Peptide LILGGSSS	Tyrosinase assay	Stomach protease hydrolysis	Kojic acid-like inhibition	–	–	–	PE	–	–	(151)
Peptide YPNPY	B16F10 cells	Competitive inhibition	–	–	Decreased melanin	–	MS	–	–	(152)
Peptides EGFG + FGDPHG	B16F10 cells + zebrafish	Dual-action	Tyrosinase inhibition	–	Antioxidant	–	MS	–	–	(153)
FUBRS	Tyrosinase assay	25–200 μg/mL	3.66 × arbutin activity	–	-	–	PE	–	–	(124)
Fermented pear juice	Pear juice	*In situ* fermentation	–	Browning degree: 1.11-2.13 → 0.48-1.64	–	–	FM	–	–	(128)
LAB pigment adsorption	Lotus rhizome juice	Fermentation	–	53.42% color increase	–	Color improvement	FM	–	–	(154)
*S. cerevisiae* extract	Fresh-cut vegetables, juice	0.5 g/L, IC_50_ 0.077 g/L	–	Browning reduced by 51.92%–102.20%	–	–	FM	–	–	(155)
*Broussonetia papyrifera* leaf fermentation	B16F10 melanoma cells	Fermented broth	Inhibited tyrosinase	–	Inhibited melanin and related protein expression	–	MS	–	–	(156)
Protective cultures (LAB)										
*L. plantarum* BX62	Fresh-cut apple	Coating, 4 °C	Significant PPO/POD inhibition	–	–	8 days (storage duration)	ST	✓	–	(129)
*L. plantarum* NML21	*Agaricus bisporus* mushroom	15 days storage	–	58.4% browning reduction	–	15 days (storage duration)	ST	–	✓	(130)
*L. plantarum* 21805 + eugenol	Fresh-cut apple	15 days at 4°C	–	Enhanced browning suppression	Consumer acceptability	15 days (storage duration)	ST	✓	–	(131)
Eugenol emulsion + *L. plantarum* EPS	Lettuce	Immersion	–	–	>3.0 log microbial reduction	>90 days stable	FM	–	✓	(132)

CA, controlled atmosphere; CMC, carboxymethyl cellulose; DPPH, 1,1-diphenyl-2-picrylhydrazyl; EGCG, epigallocatechin gallate; EMP, ethyl 3-mercaptopropionate; EPS, exopolysaccharide; FM, food matrix; FUBRS, fermented unpolished black rice sap; HPP, high-pressure processing; IC_50_, half-maximal inhibitory concentration; LAB, lactic acid bacteria; MAP, modified atmosphere packaging; MS, model system; mPPO, membrane-bound polyphenol oxidase; PE, purified enzyme; PEF, pulsed electric field; POD, peroxidase; PPO, polyphenol oxidase; SCGE, spent coffee ground extract; SMS, sodium metabisulfite; sPPO, soluble polyphenol oxidase; ST, storage trial.

### 5.7 Natural and biobased extracts

#### 5.7.1 Agro-industrial byproduct extracts

Liquid chromatography-mass spectrometry (LC-MS)/MS identified seven phenolic compounds in the pomegranate peel extract generated through US-assisted extraction. Enzyme inhibition showed that the mechanism was mixed-type, with ICs of 0.86, 0.95, and 0.73 mg mL -1 for potato, mushroom, and apple, respectively, and molecular docking indicated that potato, mushroom, and apple PPO bound tightly to the enzyme (11). These values are 10–100-fold higher than those of synthetic inhibitors such as EMP, indicating that a comparable effect is only achievable with natural extracts at much higher doses. As an alternative, fermentation was performed to increase extract potency. Yepes-Betancur et al. (120) demonstrated that fermented avocado seed extract competed with avocado PPO, as the extract completely reduced its levels.

The extract worked well at 0.5% v/v and 1.0% v/v in deferring browning. Nonetheless, it was not identified which microbial metabolites were involved in the effect on the increased activity during fermentation. This suggests a mechanistic gap: optimizing fermentation conditions rationally is difficult without knowledge of which metabolites confer activity. In another study, Galla rhois water extract was tested against apple juice, and the 250–1,000 μg/mL extract was found to be the best at preventing the 16-day browning effect. Its longer duration and greater effectiveness compared to vitamin C are promising. The sensory properties of apple juice were, however, not evaluated, and the economic value of an extract that shortens taste is limited (121). The effects of sugarcane straw phenolic extract and brewer’s spent yeast mannan extract were evaluated on fresh-cut apples. It was found that 5 g L-1 mannan extract led to greater PPO inhibition and lower loss of color, and 1 g L-1 mannan extract led to lower lipid peroxidation and loss of firmness (122). However, the effective concentration is far greater than that of most synthetic inhibitors. This makes one doubt the presence of off-odors or changes in texture at such levels. Collectively, agro-industrial byproducts can be promising long-term anti-browning agents. But effective doses need to be lowered, known active metabolites identified, sensory neutrality achieved, and seasonal stability of raw materials ensured, all of which are essential for commercial application. Through integrative control of metabolome and transcriptome pathways, vanillic acid therapy has recently been demonstrated to decrease surface browning of fresh-cut taro (123). This study demonstrates how natural phenolic compounds can simultaneously alter membrane lipid metabolism and phenolic production to produce multi-target anti-browning effects.

#### 5.7.2 Fermentation-enhanced extracts

Microbial fermentation transforms low-activity substrates into potent anti-browning extracts via specific bacteria. Sangkaew et al. (124) prepared fermented black rice using a microbial consortium consisting of *Rhizopus oryzae, Saccharomyces cerevisiae, Saccharomycopsis fibuligera*, and *Pediococcus pentosaceus.* The fermented black rice sap, after comparative analysis with the unfermented controls, exhibited much higher tyrosinase-inhibitory and antioxidant effects. The application of a known microbial consortium is a step toward controlled fermentation. Nevertheless, the precise mechanisms by which each microbe enhances bioactivity remain unclear, making it impossible to optimize fermentation conditions in a specific way.

Fermentation enhances anti-browning activity through two broad mechanisms: metabolic conversion into new bioactive compounds and microbial biotransformation that releases bound phenolics from cell wall matrices. Kim et al. (125) linked phenolic effects to tyrosinase inhibition but failed to establish a causal link. Similarly, Chang et al. (126) showed that turmeric-co-fermented mung beans perform better than vitamin C without identifying a specific contributor, and similarly reflect causal uncertainty. Unlike the mushroom tyrosinase assay by Meng et al. (127), which optimized strain selection relating to flavonoid increase with another optimization strategy, yet tested on a different cell line, the B16-F10 mouse cell, which is not a relevant cell line. In contrast to these extract-based methods, Li et al. (128) applied fermentation directly to the food product rather than using an extract. After fermenting pear juice with *Lactobacillus acidophilus* CH-2, the browning degree decreased from 1.11–2.13 to 0.48–1.64. This *in situ* fermentation model eliminates extraction steps and offers benefits for process integration. Nevertheless, since not all products may be compatible with fermented flavors, the impact on sensory properties should be considered. To sum up, fermentation-enhanced extracts have proven repeatedly useful across diverse microbial systems and substrates. However, for commercial application, identifying the exact bioactive metabolites and the biochemical pathways that produce them is required. It should be noted that some studies discussed in this section employ non-food models, such as mushroom tyrosinase assays, B16-F10 melanoma cells, and zebrafish, to evaluate tyrosinase inhibition and antimelanogenesis activity. While these models provide valuable preliminary information on the mechanistic basis of tyrosinase inhibition, they do not directly demonstrate enzymatic browning control in food matrices. Therefore, findings from these studies should be interpreted as preliminary evidence requiring validation in actual food systems before being considered equivalent to studies demonstrating anti-browning efficacy in fresh-cut products.

### 5.8 Protective cultures

#### 5.8.1 LAB as protective cultures

Lactic acid bacteria (LAB) inhibit the enzymatic browning of fruits by secreting organic acids that lower pH, competing with spoilage microbes for resources, and directly inactivating oxidative enzymes. *Lactiplantibacillus plantarum* BX62 strains were fermented in yak yogurt and used to treat fresh-cut apple slices stored at 4°C, with samples collected at 8-day intervals. *L. plantarum* BX62 slices showed reduced PPO and POD activities and lower browning compared with controls. While these results suggest a role for enzyme inhibition, the contribution of other mechanisms such as organic acid production, pH reduction, and antioxidant effects cannot be excluded. During storage, the desirable sensory attributes were maintained (129). The use of preservatives to maintain sensory quality is highly significant, as LAB metabolic activity can sometimes cause off-flavors or texture changes. This study, however, was limited to a single strain of a single product at a single concentration, thus raising questions about cross-product efficacy and optimal concentration. Shi et al. (130) tested 72 LAB strains for their ability to reduce browning in Agaricus bisporus mushrooms, replacing single-strain testing with systematic screening. The chosen strain, *L. plantarum* NML21, inhibited *Pseudomonas spp*. and decreased respiration rate and weight loss. The browning index was also reduced by 58.4% over 15 days. However, it should be noted that microbial inhibition, respiration rate, and weight loss are independent quality parameters and do not directly demonstrate anti-browning efficacy; the browning index reduction provides direct evidence of browning control. This suggests that browning inhibition is not a uniform characteristic of a LAB species but rather strain-specific. Despite the significant 58.4% decrease in browning, 41.6% of browning remains uncontrolled, suggesting that LAB may not be sufficient to prevent it entirely. In conclusion, *L. plantarum* strains BX62 and NML21 exhibit strain-specific anti-browning effectiveness in mushrooms and apples, respectively. In commercial applications, however, there is a need to ensure batch-to-batch consistency, avoid off-flavor, ensure viability under storage conditions, and incorporate into processing lines.

#### 5.8.2 LAB combined with essential oils

Combining LAB with essential oils or their components offers synergistic effects against both browning and pathogen development. Du et al. (131) compared the efficacy of eugenol and encapsulated *L. plantarum* 21805 on their half-cut apple wedges stored at 4 °C for 15 days. The co-treatment was associated with reduced physicochemical degradation, increased antioxidant enzyme activity, and improved browning suppression and consumer panel acceptability. However, the specific contribution of each component to these outcomes cannot be determined from this study design, and the mechanisms underlying the observed effects require further studies. Remarkably, customer acceptability improved as a result of browning inhibition. Combination treatments may occasionally result in unfavorable sensory changes or off-flavors that outweigh any protective benefits. Although the precise concentration ratio that improves acceptability while minimizing browning was not determined, this study indicates that eugenol and *L. plantarum* are compatible. Balyan et al. (132) demonstrated the use of the most advanced approach by showing that a 192 nm droplet-sized oil-in-water emulsion of eugenol stabilized by an exopolysaccharide produced by *L. plantarum* ATCC 8014 was obtained. The emulsion reduced the microbial load on lettuce surfaces via immersion and spray applications by >3.0 log and persisted for over 90 days under various processing conditions. The holistic approach entails deploying exopolysaccharides from the same LAB species as the protective culture. The microbe enhances formulation compatibility both directly and indirectly. But browning inhibition was not determined since this study focused on antibacterial activity. In conclusion, combinations of LAB and essential oils have synergistic potential for pathogen control and browning suppression. However, translation requires demonstrating efficacy across multiple products, consumer acceptance beyond apples, and integrating advanced encapsulation with browning-specific outcomes.

The control strategies reviewed in this section target different aspects of the browning process: membrane preservation limits enzyme-substrate contact, quinone trapping intercepts reactive intermediates, and protective cultures produce antimicrobial and antioxidant metabolites. A multi-hurdle approach combining these three mechanisms has been proposed as a conceptual framework for achieving synergistic browning control. However, it should be emphasized that no direct experimental evidence currently demonstrates synergy among these three components. The hypothesis that membrane preservation, quinone trapping, and protective cultures act synergistically to reduce browning requires validation through controlled studies that systematically evaluate their combined effects. Such studies should assess whether the combined treatment produces greater browning reduction than the sum of individual treatments, and whether the combination allows for reduced doses of individual components while maintaining efficacy. Until such evidence is available, the multi-hurdle strategy should be considered a hypothesis for future research rather than an established commercial approach. As shown in Figure 4, the eight control strategies operate through fundamentally different mechanisms: chemical inhibitors and physical methods primarily target PPO activity; atmosphere modification and edible coatings limit O_2_ availability; quinone trappers intercept o-quinones before polymerization; and protective cultures lower pH and produce inhibitory metabolites.

**FIGURE 4:**
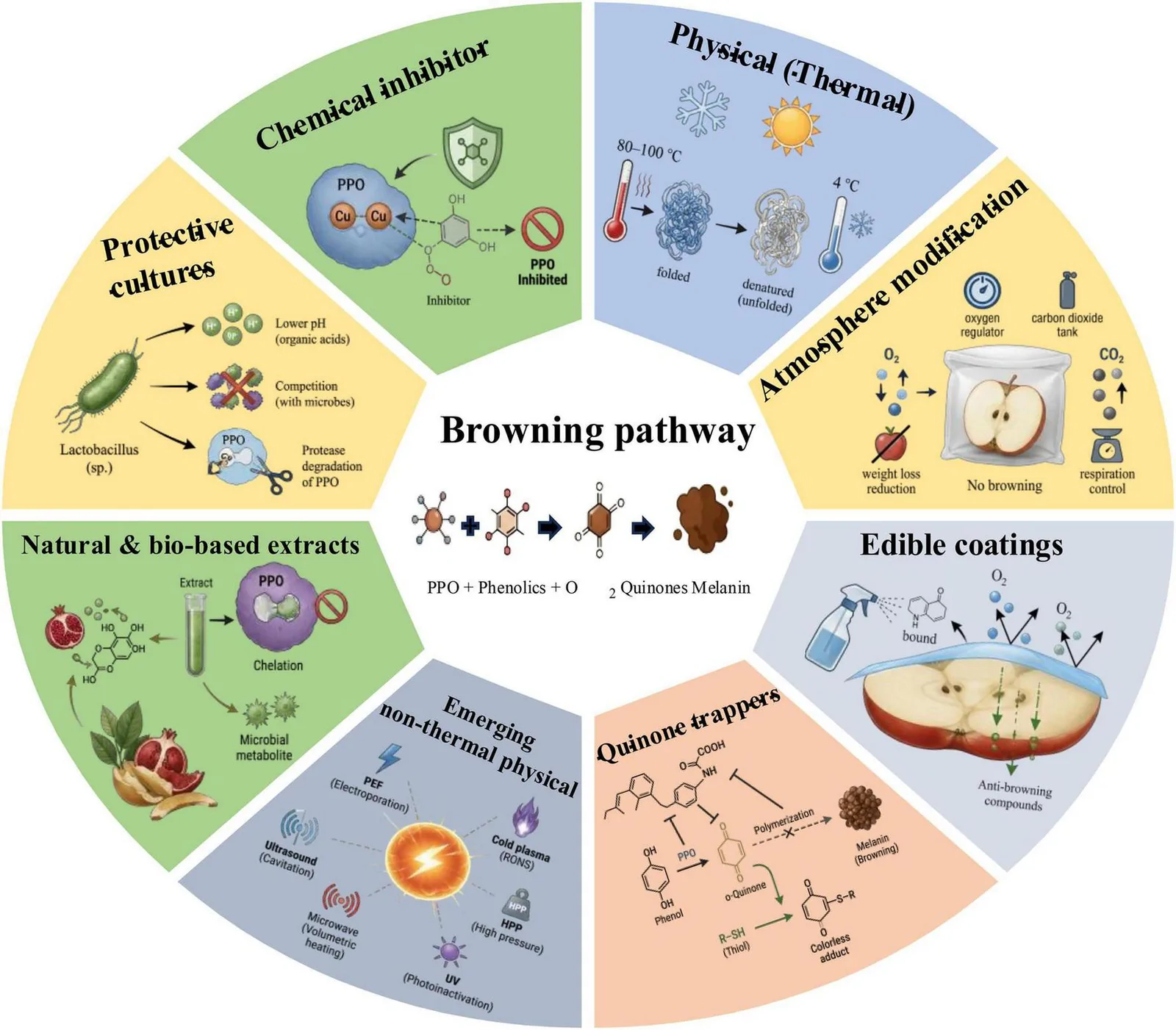
Comparative efficacy of eight control strategies for enzymatic browning inhibition in fresh-cut products.

## 6 Challenges for commercial translation and future research priorities

There are a number of interrelated challenges in the way of turning laboratory research on enzymatic browning control into commercially viable methods. While mPPO is the physiologically dominant form that causes browning in intact tissues, the extensive use of sPPO in inhibitor screening is a crucial problem (133–135). Studies consistently demonstrate that mPPO exhibits substantially higher specific activity and distinct sensitivity to inhibitors compared with sPPO, meaning that efficacy data derived from soluble enzyme preparations may not accurately predict performance in real food matrices (133, 135). Matrix effects contribute to this methodological gap because there is little correlation between *in vitro* inhibition and real browning control in foods, and the effectiveness of organic acids and other inhibitors is highly dependent on the structural characteristics of PPO and the physical properties of the food system (72). Consequently, the field requires a systematic shift toward evaluating inhibitors against mPPO and validating efficacy directly in target food matrices.

Practical factors, such as cultivar-dependent heterogeneity in browning sensitivity, sensory effects of treatments like off-flavors or texture alterations, treatment costs, energy consumption, and scalability, limit commercial translation beyond methodological considerations (136, 137). Regulatory factors are particularly crucial since, in contrast to new synthetic chemicals that need thorough toxicological testing, compounds that have already been approved as food additives or flavorings, like 3-mercapto-2-butanone, have significantly lower challenges to commercial adoption (137). Feasibility is further shaped by consumer acceptance; food technology neophobia influences willingness to buy items treated with new non-thermal technologies, and statements about shelf-life extension are less favored than health-related benefits (137, 138). Future research must therefore prioritize multi-cultivar and multi-season validation, pilot-scale and industrial testing under realistic processing conditions, systematic optimization of combined/hurdle approaches, and clear communication of health and quality benefits to consumers, thereby bridging the persistent gap between laboratory efficacy and commercial implementation.

## 7 Conclusion

Enzymatic browning involves not only enzyme-substrate reactions but also membrane-associated events that, in many physiological contexts, may precede PPO activation. PLD and LOX activity contribute to membrane degradation, facilitating enzyme-substrate contact and subsequent browning. However, the relative importance of membrane degradation versus direct enzyme-substrate contact is context-dependent, varying among commodities and browning types. Membrane degradation is not always the sole factor or mandatory initial step; in fresh-cut tissue or severe mechanical damage, decompartmentalization occurs immediately, bringing PPO, phenolics, and oxygen into direct contact. Also important is the widespread use of soluble PPO in inhibitor screening. While sPPO assays remain valuable for mechanistic screening, evidence from a limited number of comparative studies suggests they may not fully predict inhibitor performance in intact food matrices. A specific comparison using peach fruit PPO showed that ascorbic acid inhibited sPPO by 95.42% but mPPO by only 65.60%. However, this finding requires broader validation, and it should not be interpreted as a universal overestimation. Standardized screening protocols using mPPO would improve translational relevance. New detection technologies can provide early warning of browning risk, but no validated intervention protocols translate these signals into actionable responses, limiting their practical utility. A conceptual framework is proposed that links detection to risk classification, decision thresholds, intervention, and verification, but no single control strategy applies universally. A multi-hurdle strategy combining membrane preservation, quinone trapping, and protective cultures is proposed as a hypothesis requiring validation, as no direct evidence currently demonstrates synergy among these components. Future research should prioritize closed-loop detection and intervention systems, multi-cultivar and multi-season validation, and standardized mPPO-based screening protocols.
